# Plectin-mediated cytoskeletal crosstalk controls cell tension and cohesion in epithelial sheets

**DOI:** 10.1083/jcb.202105146

**Published:** 2022-02-09

**Authors:** Magdalena Prechova, Zuzana Adamova, Anna-Lena Schweizer, Miloslava Maninova, Andreas Bauer, Delf Kah, Samuel M. Meier-Menches, Gerhard Wiche, Ben Fabry, Martin Gregor

**Affiliations:** 1 Laboratory of Integrative Biology, Institute of Molecular Genetics of the Czech Academy of Sciences, Prague, Czech Republic; 2 Department of Physiology, Faculty of Science, Charles University, Prague, Czech Republic; 3 Department of Quantitative Cell Biology, Institute of Molecular Cell Biology, University of Münster, Münster, Germany; 4 Department of Physics, University of Erlangen-Nuremberg, Erlangen, Germany; 5 Department of Analytical Chemistry, University of Vienna, Vienna, Austria; 6 Department of Biochemistry and Cell Biology, Max Perutz Labs, University of Vienna, Vienna, Austria

## Abstract

The coordinated interplay of cytoskeletal networks critically determines tissue biomechanics and structural integrity. Here, we show that plectin, a major intermediate filament-based cytolinker protein, orchestrates cortical cytoskeletal networks in epithelial sheets to support intercellular junctions. By combining CRISPR/Cas9-based gene editing and pharmacological inhibition, we demonstrate that in an F-actin–dependent context, plectin is essential for the formation of the circumferential keratin rim, organization of radial keratin spokes, and desmosomal patterning. In the absence of plectin-mediated cytoskeletal cross-linking, the aberrant keratin–desmosome (DSM)–network feeds back to the actin cytoskeleton, which results in elevated actomyosin contractility. Also, by complementing a predictive mechanical model with Förster resonance energy transfer–based tension sensors, we provide evidence that in the absence of cytoskeletal cross-linking, major intercellular junctions (adherens junctions and DSMs) are under intrinsically generated tensile stress. Defective cytoarchitecture and tensional disequilibrium result in reduced intercellular cohesion, associated with general destabilization of plectin-deficient sheets upon mechanical stress.

## Teaser

Prechova et al. demonstrate that cytolinker protein plectin is essential for the formation of the circumferential keratin rim, organization of radial keratin spokes, and desmosomal patterning. Thus, plectin controls cell cohesion and maintenance of epithelial stability.

## Introduction

Epithelial tissues separate distinct compartments of organisms and face substantial mechanical stress. The mechanical integrity of epithelial sheets requires robust intercellular adhesion, which is ensured by cell–cell junctions, mainly by desmosomes (DSMs) and adherens junctions (AJs). Multiple features of junctions, such as mechanical resilience, dynamics, and signal transduction, rely on underlying cytoskeletal networks (Angulo-Urarte et al., 2020; Broussard et al., 2020; Hatzfeld et al., 2017).

In epithelial cells, the submembrane cytoskeleton consists of two circumferential structures: the actin belt (Chugh and Paluch, 2018) and the keratin rim (Quinlan et al., 2017). The actin belt associates with AJs, mediates changes in actomyosin contractility, and enables redistribution of intercellular tension (Acharya et al., 2018; Leerberg et al., 2014). Aligned parallel to cortical F-actin, the inconspicuous and hardly discernible circumferential keratin rim (Quinlan et al., 2017) remains somewhat enigmatic. The rim is associated with other keratin filaments (KFs) that are arranged into DSM-anchored radial spokes, which link nuclear and peripheral compartments of the cell. Together, these structures form an adaptable tension-spoke network (Ingber, 2003; Quinlan et al., 2017). Although multiple studies have theorized that the maintenance of epithelial homeostasis requires cooperation between actin and keratin networks (Broussard et al., 2020; Quinlan et al., 2017), our understanding of the underlying mechanisms remains fragmented.

Physical linkage of cytoskeletal systems is mediated by cytolinker proteins of the plakin protein family (Ruhrberg and Watt, 1997). Plectin (Wiche et al., 2015), a prototypical ubiquitously expressed cytolinker, has a multimodular structure that consists of a central rod domain (∼200 nm long) flanked by two globular domains. The N-terminal domain contains the canonical actin-binding domain (ABD; Andra et al., 1998), while the C-terminal repeat domains 5 and 6 harbor binding sites for intermediate filaments (IFs), thus constituting the IF-binding domain (IFBD; Nikolic et al., 1996). Plectin’s unique versatility is augmented by its transcript diversity based on different ABD-preceding sequences encoded by alternatively spliced first exons (Fuchs et al., 1999). Plectin has at least 11 known isoforms, and those most prominently expressed in epithelial tissues are plectin 1, 1a, 1c, and 1f (Fuchs et al., 1999). Besides cross-linking cytoskeletal networks, plectin can also recruit them in an isoform-specific manner to distinct structures, including the nucleus and adhesion complexes (e.g., focal adhesions and hemidesmosomes [HDs]; Rezniczek et al., 2003).

Plectin imparts mechanical stability on load-bearing tissues to maintain the tissue architecture. Mutations in the human plectin gene (*PLEC*) cause epidermolysis bullosa (EB), a disorder characterized by severe skin blistering (Rezniczek et al., 2010). In genetic mouse models, epithelia-restricted plectin ablations lead to skin fragility (Ackerl et al., 2007) or extensive intestinal lesions (Krausova et al., 2021). Mechanistically, plectin loss leads to aberrant, stress-prone keratin network organization (Jirouskova et al., 2018; Krausova et al., 2021; Osmanagic-Myers et al., 2006) in the form of more bundled and less flexible filaments (Osmanagic-Myers et al., 2006) that are uncoupled from ECM-anchored HDs (Krausova et al., 2021; Walko et al., 2011). Dysfunctional HDs lead to weakened cell adhesion, epithelial fragility, and subsequent disruption of epithelial barrier function. Although much work has focused on the role of plectin in cell–ECM adhesions (Krausova et al., 2021; Walko et al., 2011), plectin’s contribution to epithelial cell–cell cohesion remains poorly understood. Recently, an analysis of mice mimicking clinical features of familial intrahepatic cholestasis (Wu et al., 2019) revealed that DSM homeostasis and stability in biliary epithelial cells were dependent on plectin-controlled organization of KFs (Jirouskova et al., 2018). Also, we recently demonstrated that deletion of plectin in intestinal epithelial cells causes perturbations of tight junctions, AJs, and DSMs, favoring the formation of intercellular gaps in the “leaky gut” phenotype (Krausova et al., 2021).

Here, we investigate the impact of plectin inactivation on the organization of cortical cytoskeletal networks and cell–cell junctions. Our study revealed that plectin-controlled cytoarchitecture and balanced internal distribution of tension are directly linked to cell cohesion and maintenance of epithelial stability.

## Results

### Plectin defines the architecture of the epithelial KF–DSM network

**Figure 1.**
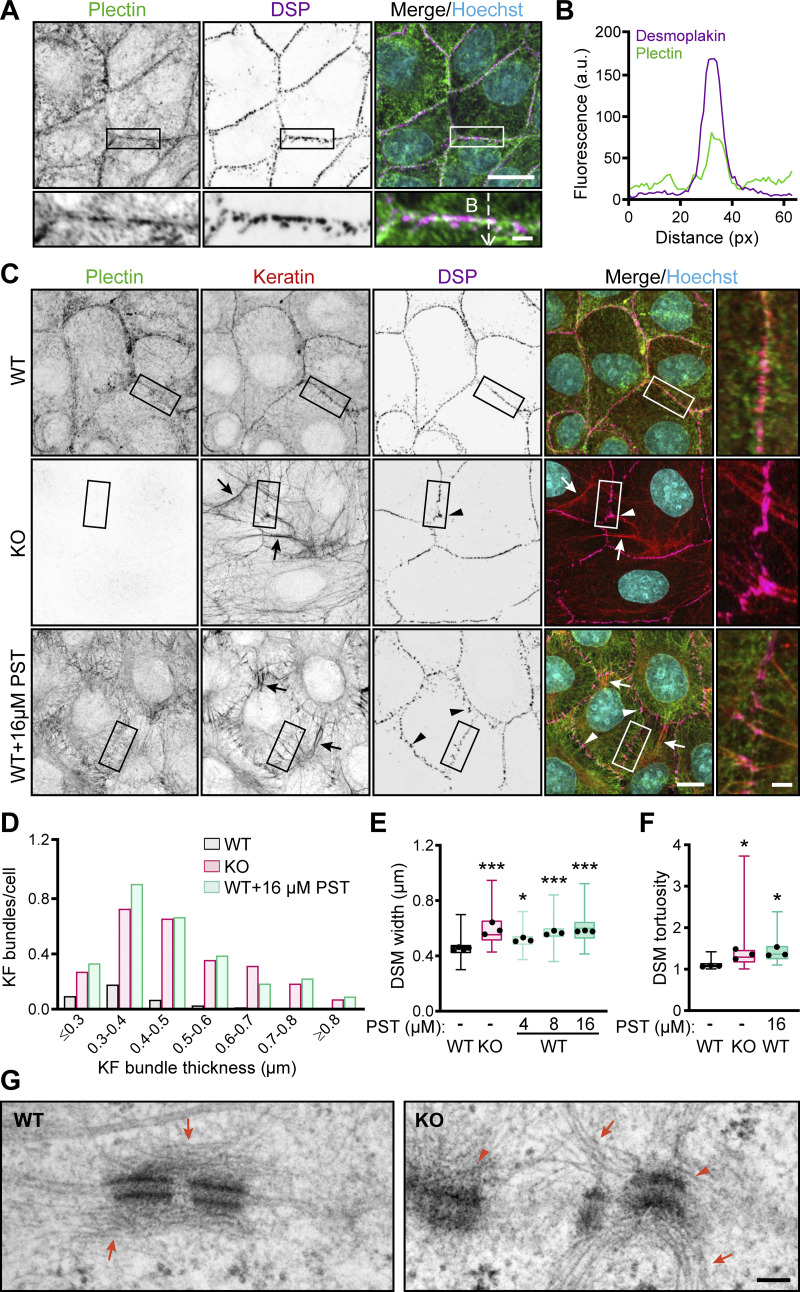
**CRISPR/Cas9- or PST-mediated plectin inhibition disrupts cytoarchitecture of KF-DSM networks in epithelial monolayers. (A)** Representative confocal images of MDCK cell monolayers immunolabeled for plectin (green) and DSP I/II (magenta). Nuclei, Hoechst (cyan). Dashed line, line scan in B. Boxed areas, ×2 images. Scale bars, 10 and 3 μm (boxed areas). **(B)** Line-scan analysis of DSP and plectin fluorescence intensity (indicated in A). **(C)** Representative confocal images of WT, KO, and PST-treated (16 μM, 4 h) WT monolayers immunolabeled for plectin (green), keratin (red), and DSP (magenta). Arrows, thick keratin bundles; arrowheads, misshaped DSMs. Boxed areas, ×2 images. Scale bars, 10 and 3 μm (boxed areas). **(D)** Histogram of the KF bundle widths from WT, KO, and PST-treated WT monolayers. *n* = 73 (WT), 70 (KO), and 54 (PST) cells; *N* = 3. **(E)** Quantification of DSM widths from WT, KO, and PST-treated WT monolayers. Boxplots show the median, 25th, and 75th percentile with whiskers reaching the last data point; dots, means of independent experiments; *n* = 135 (WT), 103 (KO), 99 (4 μM PST), 98 (8 μM PST), and 104 (16 μM PST) DSMs; *N* = 3. One-way ANOVA Tukey’s multiple comparison test; *, P < 0.05; ***, P < 0.001. **(F)** Quantification of DSM tortuosity from representative SIM images of WT, KO, and PST-treated WT monolayers. Boxplots show the median, 25th, and 75th percentile with whiskers reaching the last data point; dots, represent means of independent experiments; *n* = 74 (WT), 79 (KO), and 69 (PST) DSMs; *N* = 3. One-way ANOVA Tukey’s multiple comparison test; *, P < 0.05. **(G)** Representative TEM micrographs of DSMs in WT and KO monolayers. Arrows, KFs; arrowheads, misshaped DSMs. Scale bar, 100 nm. Source data are available for this figure: SourceData F1.

**Figure S1.**
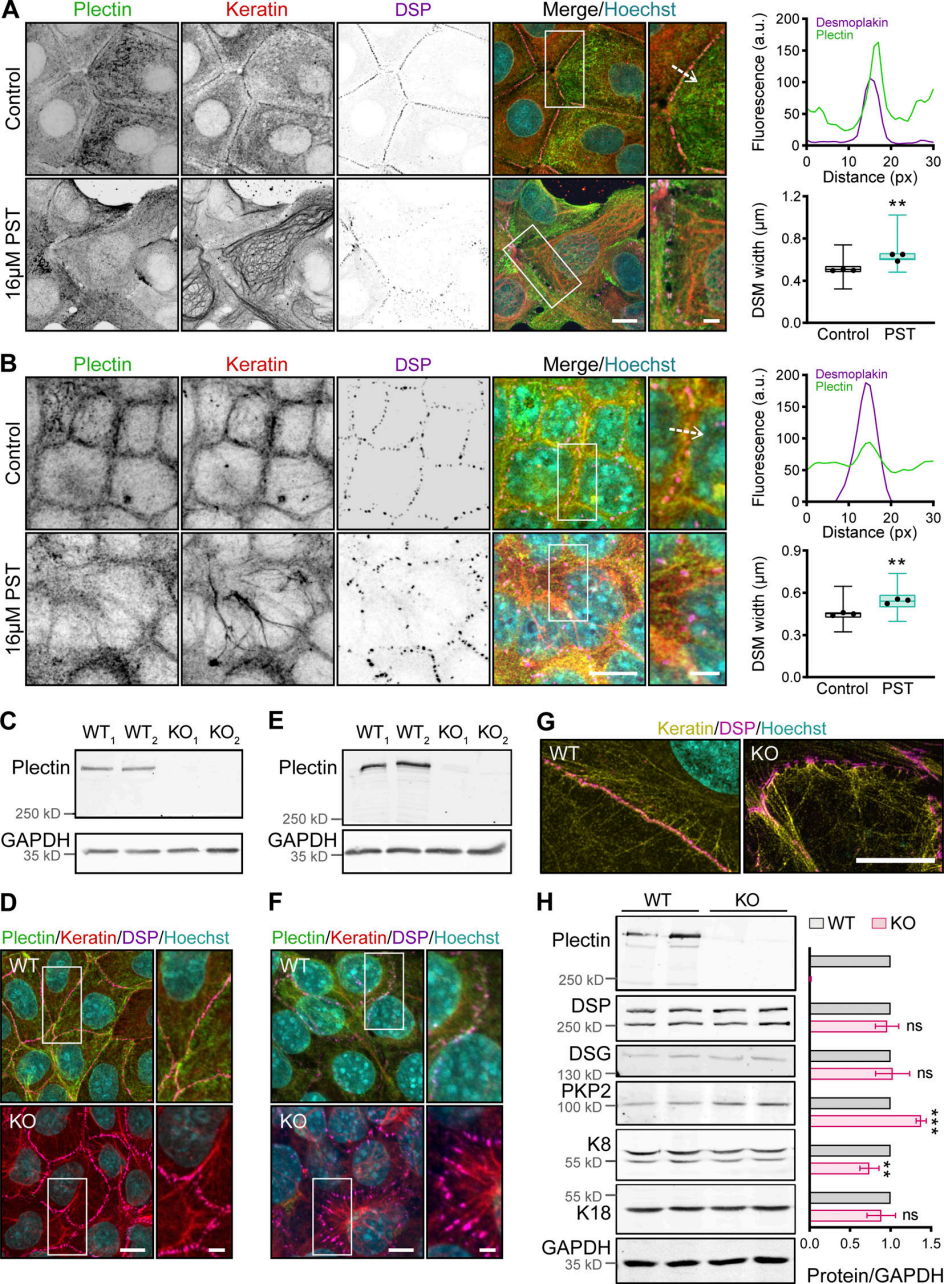
**Inactivation of plectin disrupts the cytoarchitecture of KF–DSM networks in MDCK cells, cholangiocytes, and MCF-7 cells. (A and B)** Representative confocal images of untreated (Control) and PST-treated (16 μM PST) monolayers of MCF-7 cells (A) and cholangiocytes (B) immunolabeled for plectin (green), keratin (red), and DSP (magenta). Nuclei, Hoechst (cyan). Dashed lines, line scans (upper right). Boxed areas, ×2 images. Scale bars, 10 and 3 μm (boxed areas). Graphs show line-scan analysis of DSP and plectin fluorescence intensities. Boxplots show the quantification of DSM widths (lower right). The box represents the 25th and 75th percentile with the median indicated; whiskers reach the last data point; dots, means of independent experiments; *n* (A) = 113 (Control), 93 (PST); *n* (B) = 92 (Control), and 81 (PST) DSMs; *N* = 3. Two-tailed *t* test; **, P < 0.01. **(C and E)** Immunoblots showing depletion of plectin in individual clones of KO MDCK cells (C) and cholangiocytes (E) generated with two alternative gRNAs (KO_1_ and KO_2_). **(D and F)** Representative confocal images of monolayers of WT and KO MDCK cells (D) and cholangiocytes (F) immunolabeled for plectin (green), keratin (red), and DSP (magenta). Boxed areas, ×2 images. Scale bar, 10 and 3 μm (boxed areas). **(G)** Representative SIM images of peripheral keratin structures in WT and KO MDCK monolayers immunolabeled for keratin (yellow) and DSP (magenta). Scale bar, 10 μm. **(H)** Quantification of plectin, DSP, DSG, PKP2, K8, and K18 in lysates from WT and KO monolayers by immunoblotting. GAPDH, loading control. The graph shows relative band intensities normalized to average WT values. Data are presented as mean ± SEM; *N* = 3–6. Two-tailed *t* test; **, P < 0.01; ***, P < 0.001, ns, P > 0.05. Source data are available for this figure: SourceData FS1.

Plectin has been shown to closely associate with DSM plaques in polarized MDCK cells (Eger et al., 1997). To confirm this finding, we inspected the intracellular localization of plectin in epithelial monolayers grown from MDCK cells, mouse cholangiocytes, and epithelial breast cancer cells (MCF-7) using immunofluorescence microscopy. In all three cell lines, plectin clearly delineated cell–cell borders, where it closely colocalized with the DSM marker desmoplakin (DSP; Fig. 1, A and B and Fig. S1, A and B).

To explore the role of plectin in epithelial sheets, we generated plectin-deficient (KO) MDCK cells and cholangiocytes using the CRISPR/Cas9 system (Jirouskova et al., 2018). We confirmed the successful ablation of plectin by immunofluorescence microscopy and immunoblot analysis (Fig. S1, C–F). We also treated cell monolayers with the small organoruthenium-based compound plecstatin-1 (PST), a high-affinity plectin ligand that exhibits inhibitory effects (Meier et al., 2017). As plectin is a major organizer of epithelial KFs (Jirouskova et al., 2018; Krausova et al., 2021; Osmanagic-Myers et al., 2006), we inspected the keratin cytoarchitecture in these monolayers by immunofluorescence microscopy. In untreated monolayers, KFs typically formed a network where a subset of filaments aligned into dense radial spokes spanning the cytoplasmic space between the nucleus and DSP-positive DSMs, the latter often resembling beads on a string (Fig. 1 C). The rim-and-spoke KF configuration was clearly discernible when super-resolution structured illumination microscopy (SIM) was employed (Fig. S1 G).

Genetic and pharmacological plectin inactivation produced less delicate keratin networks with radial filaments bundled into sparse, thickened spokes (Fig. 1 C and Fig. S1, A and B). The spokes were tethered to less dense, wider, and contorted DSMs at the cell–cell borders. This phenotype was independent of the gRNA sequences used for plectin depletion (Fig. S1, D and F) and was also confirmed in PST-treated cholangiocytes and MCF-7 cells (Fig. S1, A and B). The changes in KF-DSM organization probably were not a consequence of altered protein expression levels, as plectin inactivation affected only the expression of plakophilin-2 and keratin 8 (K8), but not that of DSP, desmoglein 1/2 (DSG), and keratin 18 (K18; Fig. S1 H).

A quantitative analysis of the KF–DSM network morphology confirmed frequent formation of significantly thicker keratin bundles in monolayers with disabled plectin (Fig. 1 D). When we quantified the DSM cross-sectional width (measured as outer plaque-to-plaque distance), we found that DSMs were significantly dilated upon genetic and pharmacological plectin targeting (Fig. 1 E). Also, higher tortuosity (calculated as the ratio between the actual and the linear length) indicated highly irregular and misshaped DSMs (Fig. 1 F). Treatment with 16 μM PST yielded phenotypes comparable to plectin ablation (Fig. 1, D–F), whereas 4 and 8 μM PST produced intermediate phenotypes (Fig. 1 E).

To analyze structural features of KFs and DSMs in more detail, we compared their ultrastructure in monolayer cultures of WT and KO MDCK cells, using transmission EM (TEM). Micrographs of KO cells revealed frequently irregular, prominently widened DSMs with seemingly dilated intercellular spaces between adjacent cells (Fig. 1 G). At the same time, individually resolved KFs in KO monolayers were no longer aligned parallel to the membrane, while prominent KF bundles extended from the cell interior towards the widened DSMs. These alterations confirmed the phenotypes observed by immunofluorescence microscopy. Hence, using gene editing and a pharmacological approach, we showed that plectin is essential for the architecture of KF–DSM networks in epithelial monolayers.

### Plectin facilitates an adaptive cellular response to mechanical stress to sustain cell cohesion in epithelial sheets

**Figure 2.**
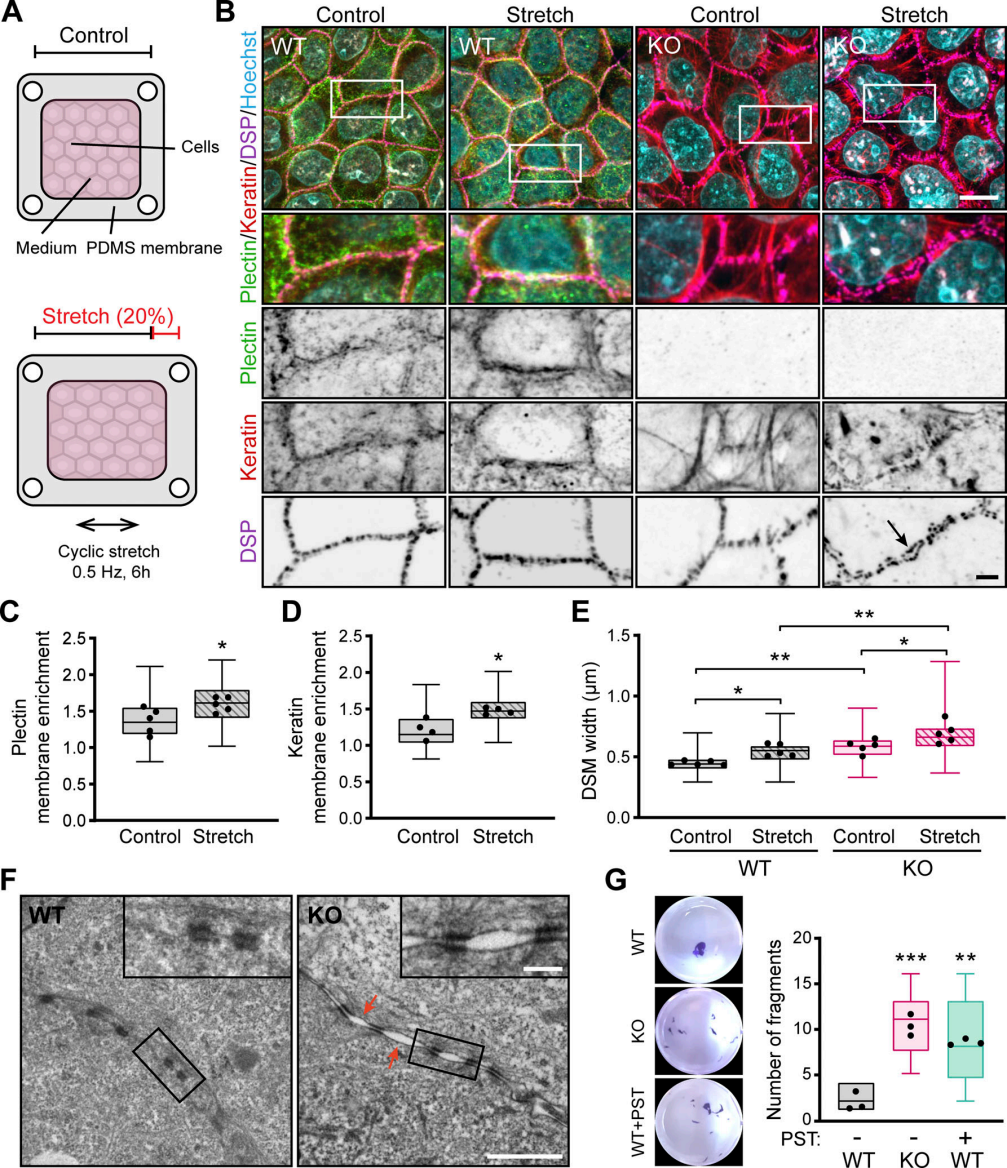
**Plectin deficiency renders the monolayers incapable of effectively adapting to mechanical stress. (A)** Schematics of the monolayer stretching experimental setup. **(B)** Representative confocal images of unchallenged (Control) or stretched (Stretch) monolayers from WT and KO MDCK cells immunolabeled for plectin (green), keratin (red), and DSP (magenta). Nuclei, Hoechst (cyan). Arrow, dilated DSM. Boxed areas, ×2 images. Scale bars, 10 and 3 μm (boxed areas). **(C and D)** Quantification of plectin (C) and keratin (D) relative membrane enrichment, calculated as a ratio of fluorescence intensity at cell–cell borders and a total fluorescence intensity per cell from monolayers shown in B. Boxplots show the median, 25th, and 75th percentile with whiskers reaching the last data point; dots, means of independent experiments; *n* (C) = 89 (Control) and 112 (Stretched); *n* (D) = 80 (Control) and 105 (Stretched) cell–cell borders; *N* = 5 (C), 4 (D). Two-tailed *t* test; *, P < 0.05. **(E)** Quantification of DSM widths from control and stretched monolayers. Boxplots show the median, 25th, and 75th percentile with whiskers reaching the last data point; dots, means of independent experiments; *n* = 170 (WT Control), 167 (WT Stretched), 158 (KO Control), 153 (KO Stretched) DSMs; *N* = 5. One-way ANOVA Tukey’s multiple comparison test; *, P < 0.05; **, P < 0.01. **(F)** Representative TEM images of DSMs in stretched WT and KO monolayers. Arrows, dilated intercellular spaces. Boxed areas, ×4 images. Scale bars, 1 μm and 200 nm (boxed areas). **(G)** Fragmented WT, KO, and PST-treated (16 μM) monolayers after dispase-based mechanical dissociation assay. Boxplots show the median, 25th, and 75th percentile with whiskers reaching the last data point; dots, means of independent experiments; *n* = 10 (WT), 9 (KO) and 6 (WT + PST); *N* = 3. One-way ANOVA Tukey’s multiple comparison test; **, P < 0.01; ***, P < 0.001.

**Figure S2.**
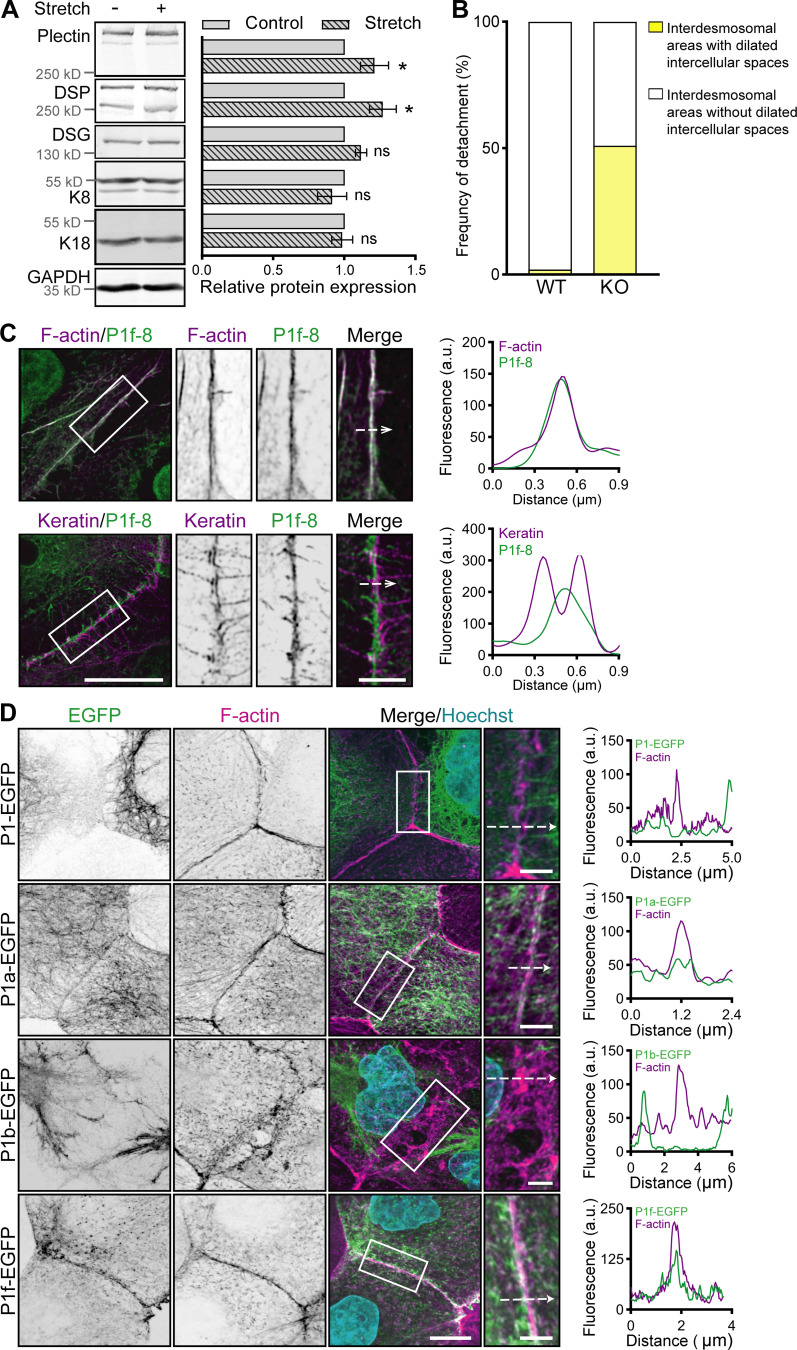
**Stretch-induced changes, expression of P1f-8-EGFP and NCD treatment in cell monolayers. (A)** Quantification of plectin, DSP, DSG, K8, and K18 in lysates from WT and KO MDCK monolayers by immunoblotting. GAPDH, loading control. The graph shows relative band intensities normalized to average WT values. Data are presented as mean ± SEM; *N* = 4. Two-tailed *t* test; *, P < 0.05; ns, P > 0.05. **(B)** Graph shows the percentage of interdesmosomal areas with dilated intercellular spaces in monolayers shown in Fig. 2 F. *n* = 164 (WT) and 167 (KO) interdesmosomal areas. **(C)** Representative STED images of peripheral F-actin (upper panel) and keratin (lower panel) structures in WT MDCK monolayers expressing EGFP-tagged plectin fragment 1f-8 (P1f-8; green) and stained for F-actin and keratin (both magenta). Dashed lines, line scans (right). Boxed areas, ×2 images. Scale bar, 10 and 3 μm (boxed areas). Graphs show line-scan analysis of P1f-8-EGFP and F-actin (upper panel) and keratin (lower panel) fluorescence intensities. **(D)** Representative confocal images of midsections of KO MDCK monolayers expressing indicated full-length EGFP-tagged plectin isoforms (green) and stained for F-actin (magenta). Nuclei, Hoechst (cyan). Dashed lines, line scans (right). Boxed areas, ×2 (P1b) and ×2.66 (P1, P1a, and P1f) images. Scale bars, 10 and 3 μm (boxed areas). Graphs show line-scan analysis of indicated full-length EGFP-tagged plectin isoforms and F-actin fluorescence intensities. Source data are available for this figure: SourceData FS2.

Recent work implies that plectin mediates the cellular response to cholestasis (Jirouskova et al., 2018), which provides the biliary epithelium with resilience against increased bile pressure. To investigate whether plectin is involved in active epithelial adaptation to mechanical perturbation, we exposed MDCK cell monolayers to cyclic, uniaxial mechanical stretch (Kah et al., 2021). In line with our previous report (Jirouskova et al., 2018), 6 h of continuous 20% stretch (Fig. 2 A) triggered higher expression levels of plectin and DSP, whereas expression of DSG and keratins (K8 and K18) remained comparable to that of unstretched (control) conditions (Fig. S2 A).

Inspection of WT monolayers after stretching revealed a relocalization of plectin from the cytoplasmic pool towards cell–cell borders (Fig. 2 B), as confirmed by quantitative fluorescence microscopy (Fig. 2 C). The stretch-induced recruitment of plectin to the plasma membrane was associated with a remodeling of the keratin network. This was manifested as an enrichment of KFs along the plasma membrane and their coalignment with plectin-positive DSMs (Fig. 2, B and D). As predicted, given the changes in the KF–DSM architecture upon plectin inactivation, KF networks in unstretched KO cell monolayers were found to be less delicate and disarrayed compared with WT monolayers (Fig. 2 B).

The stretch-induced reorganization of KFs in KO sheets was accompanied by a prominent disorganization of DSMs (Fig. 2 B). Consistent with previous studies (Felder et al., 2008; Lutz et al., 2020), our quantitative analysis revealed that monolayer stretching led to a significant increase in the cross-sectional DSM width in both WT and KO monolayers (Fig. 2 E). Moreover, DSMs were often dramatically dilated, with plaques sometimes >1 μm apart (Fig. 2 E), indicating that the monolayers were stretched to their limits. This was also confirmed by TEM analysis, which showed that despite the remaining DSM connections, expanded intercellular spaces occurred frequently between neighboring KO cells (Fig. 2 F and Fig. S2 B).

To confirm the role of plectin in maintaining epithelial sheet integrity, we assessed intercellular adhesion strength in monolayers using a dispase-based mechanical dissociation assay. Consistent with epithelial disruption upon stretch, both KO- and PST-treated WT sheets generated a significantly higher number of fragments after shear stress when compared with WT counterparts (Fig. 2 G). These results collectively show that plectin is essential for strain-induced reorganization of keratin networks and reinforcement of cell cohesion, both of which are required for effective epithelial mechanoprotection.

### Plectin controls the formation of a circumferential keratin rim in an isoform-specific manner

**Figure 3.**
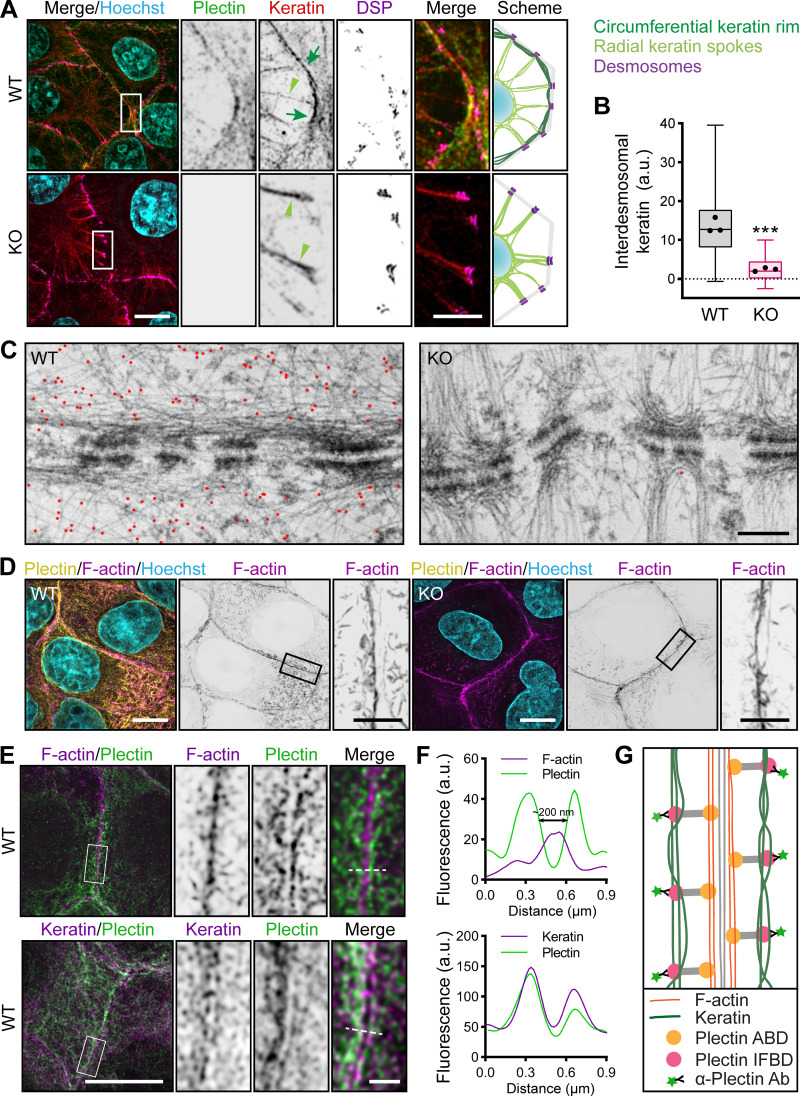
**Plectin is required for circumferential keratin rim formation. (A)** Representative SIM images of peripheral keratin structures in WT and KO MDCK monolayers immunolabeled for plectin (green), keratin (red), and DSP (magenta). Nuclei, Hoechst (cyan). Arrows, circumferential keratin rim; arrowheads, keratin spokes. Note the absence of the keratin rim signal in the KO monolayer. Boxed areas, ×4 images. Scale bars, 10 and 3 μm (boxed areas). Drawn schematics depict the rim-and-spoke keratin configuration in WT (upper panel) and KO (lower panel) cells. **(B)** Quantification of interdesmosomal keratin fluorescence intensity, corrected for background intensity (dashed line). Boxplots show the median, 25th, and 75th percentile with whiskers reaching the last data point; dots, means of independent experiments; *n* = 188 (WT) and 100 (KO) interdesmosomal areas; *N* = 3. Two-tailed *t* test; ***, P < 0.001. **(C)** Representative TEM images of DSMs at cell–cell borders in WT and KO monolayers immunolabeled with 10-nm gold particle (red pseudocolor)–conjugated antibodies against plectin. Scale bar, 200 nm. **(D)** Representative SIM images of cortical F-actin in apical section of WT and KO monolayers stained for plectin (yellow) and F-actin (magenta). Boxed areas, ×4 images. Scale bars, 10 and 3 μm (boxed areas). **(E)** Representative STED images of cortical keratin and actin structures in WT monolayers stained for plectin (green) and F-actin (magenta; upper panels) and keratin (magenta; lower panels). Dashed lines, line scans in F. Boxed areas, ×4 images. Scale bar, 10 and 1 μm (boxed areas). **(F)** Line-scan analysis of plectin and F-actin (upper panel) or keratin (lower panel) fluorescence intensities (indicated in E). **(G)** Schematic representation depicts submembranous organization of plectin, F-actin, and KFs.

Mechanically robust cell cohesion in epithelial cells relies on subplasmalemmal cytoskeletal structures, which provide physical support to the plasma membrane and intercellular junctions (Angulo-Urarte et al., 2020; Broussard et al., 2020; Hatzfeld et al., 2017). Given the reduced mechanical resilience of plectin KO MDCK cell sheets, we compared the appearance of circumferential keratin and actin networks in WT and KO monolayers using SIM. Images of WT monolayers showed a clearly discernible rim of interdesmosomal KFs, with keratin spokes extending from DSMs towards the cell interior (Fig. 3 A). In sharp contrast, no filamentous keratin juxtapositioned to the borders of confluent KO cell sheets was found and only sparse, irregularly distributed DSMs with widely separated prominent radial spokes were observed (Fig. 3, A and B). Confirming this observation, TEM using plectin-specific immunogold labeling (Fig. 3 C) revealed long sections of unsupported cell membranes in between distorted DSMs. Remarkably, in both WT and KO monolayers, cortical actin filament organization appeared unaffected (Fig. 3 D).

The unexpected absence of the circumferential keratin rim in KO epithelial sheets prompted us to analyze the relative localization of WT subplasmalemmal cytoskeletal networks using super-resolution stimulated emission depletion (STED) microscopy. In line with previous findings (Chugh and Paluch, 2018), cortical F-actin formed a thick circumferential belt closely aligned with the cell borders that were so close that the signal contributions from two neighboring cells could not be clearly resolved (Fig. 3, E and F). By contrast, two individual keratin fluorescence line-scan peaks were discernible (Fig. 3, E and F), corresponding to distinct keratin rims (one contributed by each cell). These rims coaligned with the cell–cell borders at a distance of ∼200 nm from the cortical F-actin (Fig. 3, E and F). Interestingly, plectin staining evenly decorated both sides of F-actin structures and colocalized with circumferential keratin (Fig. 3, E and F). As plectin was visualized with antibodies that recognize its C-terminus, we hypothesized that plectin, by virtue of its structure, could simultaneously interact with keratin (via C-terminal IFBD) and cortical F-actin (via N-terminal ABD; Fig. 3 G). Consistent with this notion, we found perfect colocalization between cortical F-actin and an ectopically expressed N-terminal fragment of plectin, comprising just its isoform-specific (exon 1f) sequence and the succeeding ABD, fused to EGFP (P1f-8-EGFP; Fig. S2 C).

**Figure 4.**
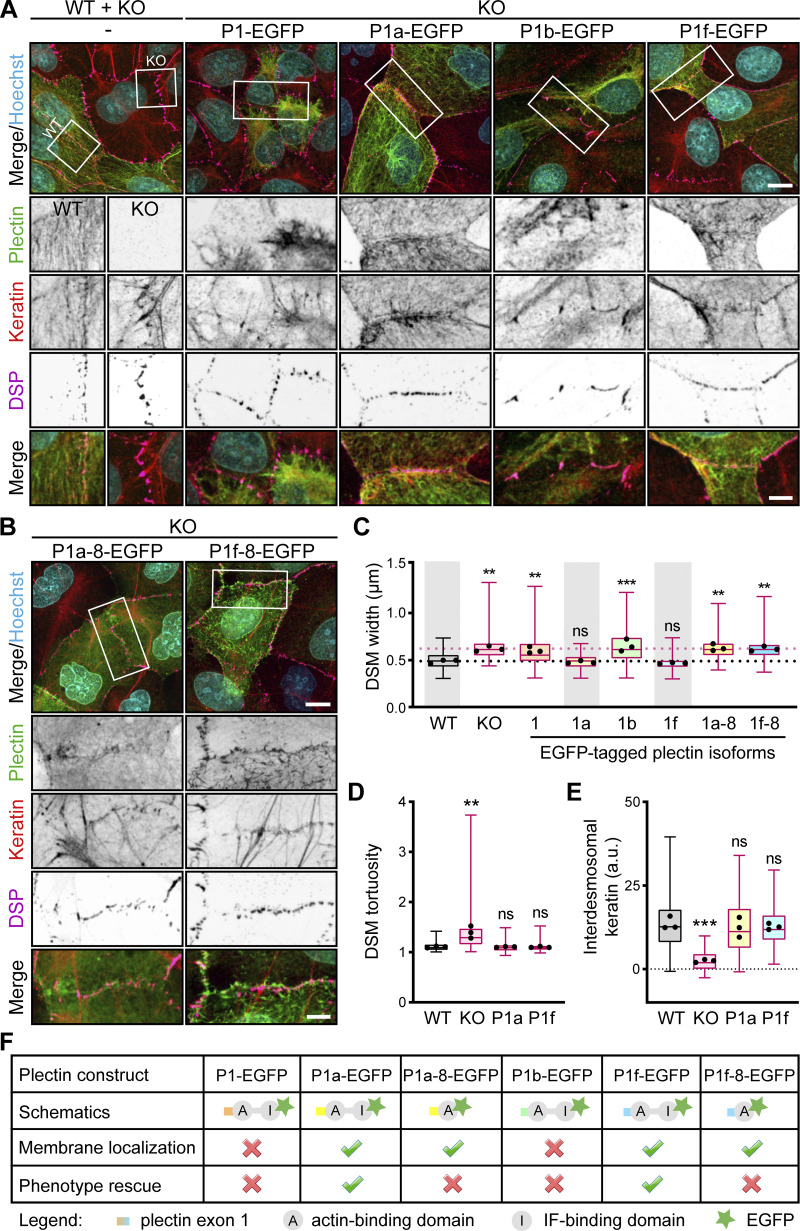
**Re-expression of plectin isoforms P1f and P1a rescues circumferential keratin rim formation and restores DSM morphology. (A)** Representative confocal images of monolayers formed by a mixed population of WT and KO MDCK cells (WT + KO) immunolabeled for plectin (green), keratin (red), and DSP (magenta; left), and KO monolayers transfected with indicated full-length EGFP-tagged plectin isoforms (green) and immunolabeled for keratin (red) and DSP (magenta). Nuclei, Hoechst (cyan). Boxed areas, ×2 images. Scale bar, 10 and 5 μm (boxed areas). **(B)** Representative confocal images of KO monolayers transfected with indicated EGFP-tagged plectin fragments (green) and immunolabeled for keratin (red) and DSP (magenta). Boxed areas, ×2 images. Scale bar, 10 and 5 μm (boxed areas). **(C)** Quantification of DSM widths from monolayers shown in A and B. Boxplots show the median, 25th, and 75th percentile with whiskers reaching the last data point; dots, means of independent experiments; *n* = 107 (WT), 126 (KO), 120 (P1), 127 (P1a), 80 (P1b), 109 (P1f), 81 (P1a-8), and 110 (P1f-8) DSMs; *N* = 3. One-way ANOVA Tukey’s multiple comparison test; **, P < 0.01; ***, P < 0.001, ns, P > 0.05. **(D)** Quantification of DSM tortuosity from SIM images of mixed WT and KO cells and indicated isoforms re-expressing KO monolayers. Boxplots show the median, 25th, and 75th percentile with whiskers reaching the last data point; dots, means of independent experiments; *n* = 74 (WT), 79 (KO), 92 (P1a), and 73 (P1f) DSMs; *N* = 3. One-way ANOVA Tukey’s multiple comparison test; **, P < 0.01; ns, P > 0.05. **(E)** Quantification of interdesmosomal keratin fluorescence intensity, corrected for background intensity (dashed line). Boxplots show the median, 25th, and 75th percentile with whiskers reaching the last data point; dots, means of independent experiments; *n* = 142 (WT), 101 (KO), 144 (P1a), and 104 (P1f) interdesmosomal areas; *N* = 3. One-way ANOVA Tukey’s multiple comparison test; ***, P < 0.001; ns, P > 0.05. **(F)** Summary of plectin-based constructs re-expression in plectin KO cells.

**Figure S3.**
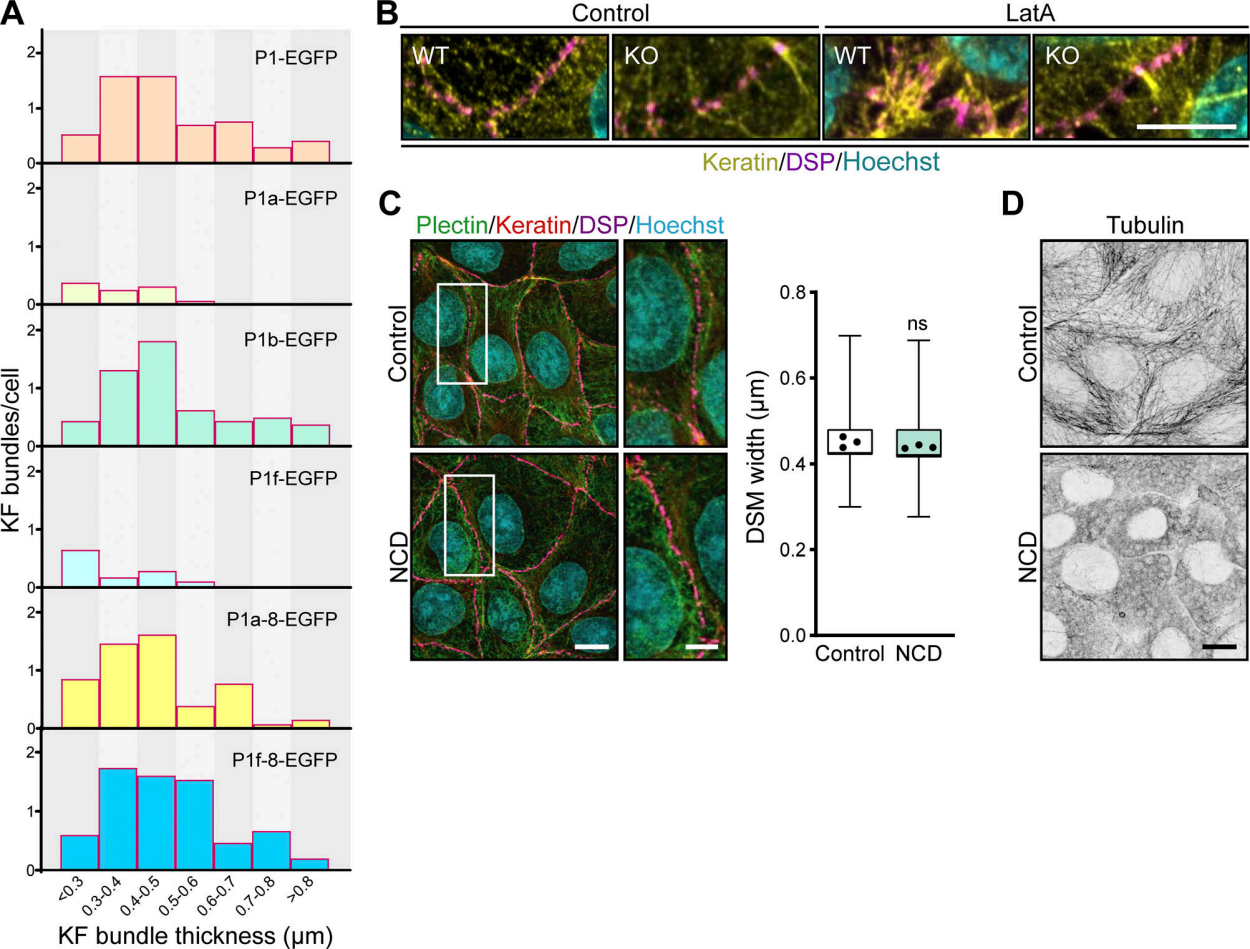
**Re-expression of EGFP-tagged plectin isoforms and LatA treatment in cell monolayers.** **(A)** Histogram of KF bundle widths from KO monolayers expressing indicated full-length EGFP-tagged plectin isoforms. *n* = 18 (P1), 16 (P1a), 16 (P1b), 28 (P1f), 13 (P1f-8), and 15 (P1a-8) cells; *N* = 3. **(B)** Confocal images of untreated (Control) and LatA-treated (1 μM, 30 min) WT and KO monolayers stained for keratin (yellow) and DSP (magenta). Nuclei, Hoechst (cyan). Scale bar, 10 μm. **(C)** Representative confocal images of untreated (Control) and NCD-treated (10 μM, 1 h) WT cells immunolabeled for plectin (green), keratin (red), and DSP (magenta). Boxed areas, ×2 images. Scale bar, 10 and 5 μm (boxed areas). The boxplot shows the quantification of DSM widths. The box represents the 25th and 75th percentile with the median indicated; whiskers reach the last data point; dots, means of independent experiments; *n* = 90 (Control) and 72 (NCD) DSMs; *N* = 3. Two-tailed *t* test; ns, P > 0.05. **(D)** Representative confocal images of control and NCD-treated WT monolayers immunolabeled for tubulin. Note efficient depolymerization of microtubules upon NCD treatment. Scale bar, 10 μm.

To demonstrate that the observed changes in KF–DSM networks were directly connected to plectin deficiency, the phenotypic rescue potential of plectin was examined by transient transfection of MDCK KO cells with expression plasmids encoding full-length, EGFP-tagged plectin isoforms, which were previously found to be expressed in epithelia (Fuchs et al., 1999). To this end, two plectin isoforms implicated in IF organization, P1a (Osmanagic-Myers et al., 2006; P1a-EGFP) and P1f (Burgstaller et al., 2010; P1f-EGFP), and two organelle-associated isoforms, P1 (nucleus [Staszewska et al., 2015]; P1-EGFP) and P1b (mitochondria [Winter et al., 2008]; P1b-EGFP) were tested (Fig. 4 A). Like endogenous plectin of the corresponding WT cells (Fig. 3, D and E), re-expressed P1a- and P1f-EGFP closely coaligned with cortical F-actin delineating cell–cell borders (Fig. S2 D). By contrast, P1- and P1b-EGFP were not enriched along membranes and mainly remained distributed throughout the cytoplasm (Fig. S2 D). Immunofluorescence microscopy showed that KO cells expressing either P1a- or P1f-EGFP, but not P1- and P1b-EGFP, displayed nearly complete restoration of the circumferential keratin rim (Fig. 4, A and E). A quantification of the KF–DSM phenotype revealed that DSM width/tortuosity and keratin bundle thickness in P1a- and P1f-transfected cells were similar to the values found in WT cells (Fig. 4, C and D; Fig. S3 A; and Fig. 1, D–F).

To examine whether restoration of KF and DSM organization required the expression of full-length P1a and P1f, in particular, of its C-terminal IFBD (Nikolic et al., 1996), we transfected KO cells with truncated P1a- and P1f-8-EGFP cDNA constructs. Subsequent analysis showed that neither P1a-8- nor P1f-8-EGFP, albeit both being targeted to the cell periphery (Fig. 4 B and Fig. S2 C), were able to restore the WT phenotype (Fig. 4, B and C and Fig. S3 A). The strong rescue potential of full-length, but not truncated, membrane-enriched plectin isoforms P1a and P1f clearly demonstrates a direct link between the absence of plectin and the observed alterations of the KF–DSM architecture (Fig. 4 F). Together, these data suggest that plectin, in an isoform-specific manner, secures the formation of the circumferential keratin rim and possibly integrates keratin and actin subplasmalemmal networks to provide mechanical support to the plasma membrane and cell–cell contacts.

### Formation of the circumferential keratin rim requires plectin interaction with cortical F-actin

**Figure 5.**
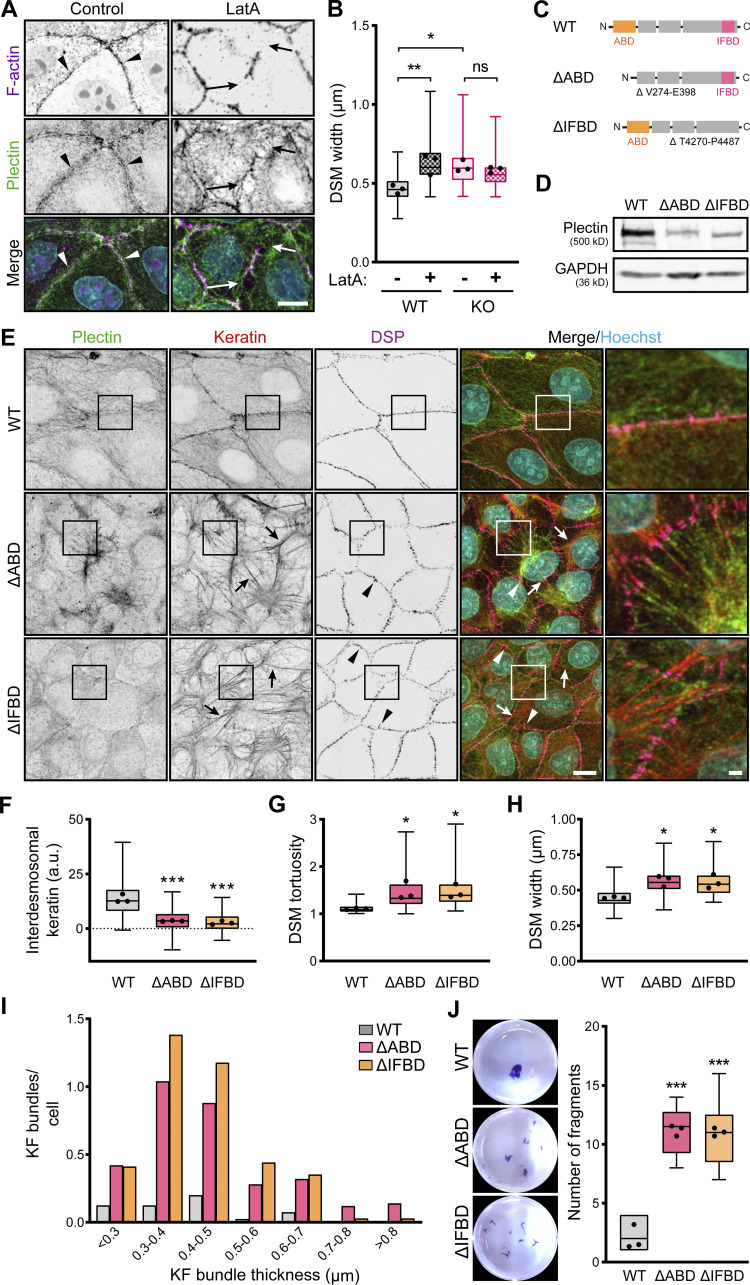
**Intact KF–DSM networks require plectin interaction with both F-actin and KFs. (A)** Representative confocal images of untreated (Control) and LatA-treated (1 μM, 30 min) WT MDCK monolayers stained for plectin (green) and F-actin (magenta). Nuclei, Hoechst (cyan). Arrowheads, regions of plectin and cortical F-actin colocalization; arrows, regions of depolymerized cortical F-actin. Note relocalization of plectin from the plasma membrane in the absence of cortical F-actin. Scale bar, 10 μm. **(B)** Quantification of DSM widths from indicated WT and KO monolayers shown in A. Boxplots show the median, 25th, and 75th percentile with whiskers reaching the last data point; dots, means of independent experiments; *n* = 100 (WT), 123 (WT + LatA), 114 (KO), and 109 (KO + LatA) DSMs; *N* = 3. One-way ANOVA Tukey’s multiple comparison test; *, P < 0.05; **, P < 0.01; ns, P > 0.05. **(C)** Schematic of CRISPR/Cas9-engineered deletions between amino acid residues V274-E398 or T4270-P4487 (Uniprot accession no. F1PHS5), corresponding to actin (∆ABD; orange) and the IF (∆IFBD; pink) binding domains (for details, see Fig. S4 A and Materials and methods section). **(D)** Immunoblot analysis of mutated MDCK clones harboring ∆ABD and ∆IFBD deletion. GAPDH, loading control. **(E)** Representative confocal images of WT, ∆ABD, and ∆IFBD monolayers immunolabeled for plectin (green), keratin (red), and DSP (magenta). The same images are also presented in Fig. S4. Arrows, thick keratin bundles; arrowheads, misshaped DSMs. Boxed areas, ×2 images. Scale bar, 10 and 3 μm (boxed areas). **(F–H)** Quantification of interdesmosomal keratin fluorescence intensity (F), DSM tortuosity (G) and widths (H) from SIM (F and G) and confocal (H) images of WT, ∆ABD, and ∆IFBD monolayers. Boxplots show the median, 25th, and 75th percentile with whiskers reaching the last data point; dots, means of independent experiments; *n* (F) = 142 (WT), 94 (∆ABD), and 99 (∆IFBD) interdesmosomal areas; *n* (G) = 74 (WT), 65 (∆ABD), and 70 (∆IFBD) DSMs; *n* (H) = 102 (WT), 102 (∆ABD), and 93 (∆IFBD) DSMs; *N* = 3. One-way ANOVA Tukey’s multiple comparison test; *, P < 0.05; ***, P < 0.001. **(I)** Histogram of the KF bundle widths from WT, ∆ABD, and ∆IFBD monolayers. *n* = 40 (WT), 50 (∆ABD), and 34 (∆IFBD) cells; *N* = 3. **(J)** Fragmented WT, ∆ABD, and ∆IFBD monolayers after dispase-based mechanical dissociation assay. Boxplots show the median, 25th, and 75th percentile with whiskers reaching the last data point; dots, means of independent experiments; *N* = 3. One-way ANOVA Tukey’s multiple comparison test; ***, P < 0.001. Source data are available for this figure: SourceData F5.

To investigate whether cortical F-actin is required for submembranous localization of plectin, we treated WT monolayers with low doses of the actin-depolymerizing drug latrunculin A (LatA). Exposure to LatA led to local disruption of the actin belt, while most of the belt remained preserved along cell–cell borders (Fig. 5 A). Intriguingly, in the regions where F-actin was depolymerized, plectin lost its membranous enrichment and redistributed towards the cytoplasm. Moreover, LatA treatment led to keratin bundling and widening of DSMs in WT monolayers (Fig. 5 B and Fig. S3 B), closely mimicking the situation in untreated KO monolayers (Fig. 1, C and E). These effects were specific to perturbation of the actin cytoskeleton, as nocodazole (NCD) treatment, which depolymerizes microtubules, did not affect the keratin network or the DSM width (Fig. S3, C and D). Overall, these data suggest that plectin recruitment to peripheral cytoskeletal structures is F-actin dependent.

**Figure S4.**
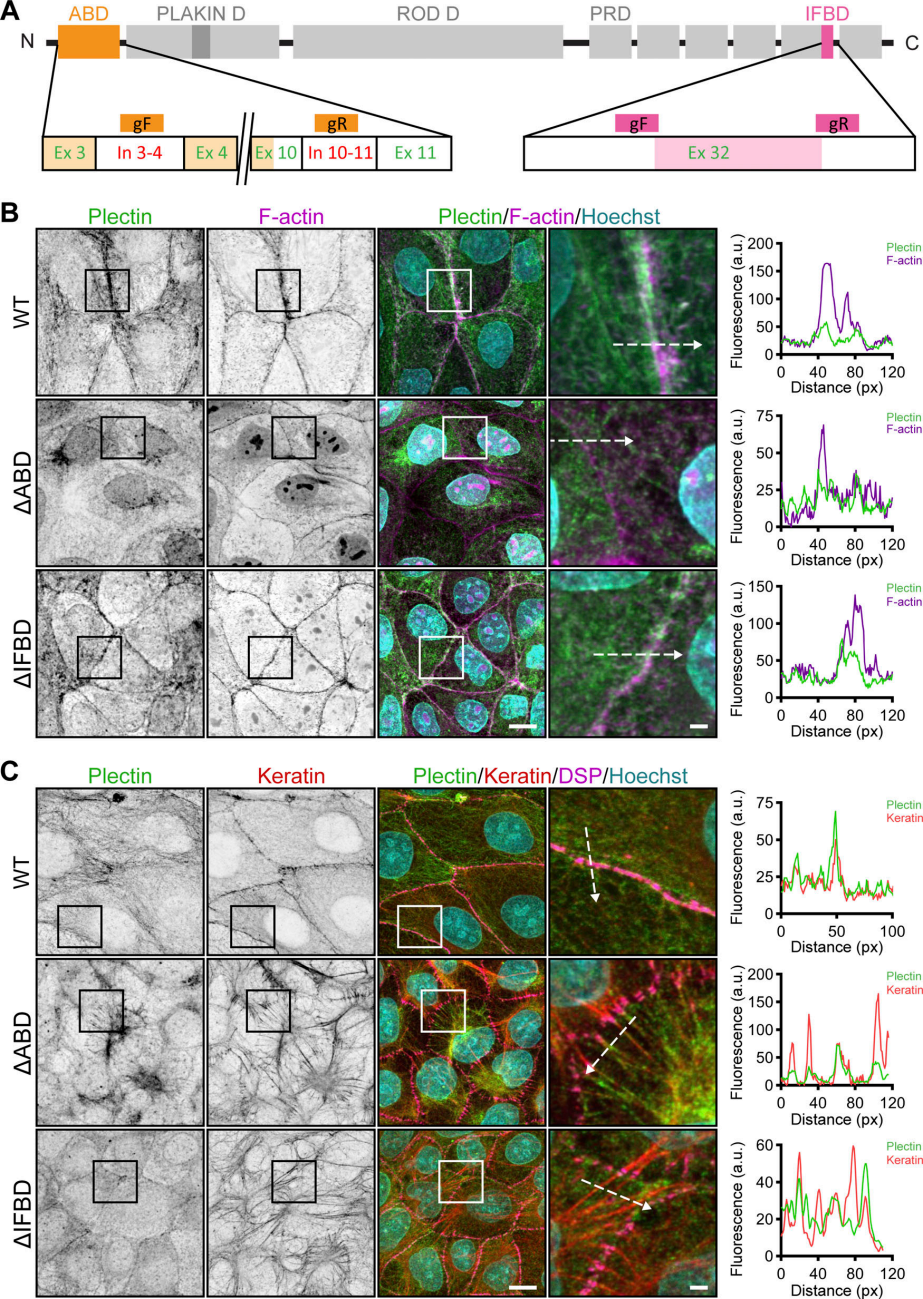
**CRISPR/Cas9-mediated ablation of actin and IF binding domains. (A)** Schematic representation of CRISPR/Cas9-engineered deletions between amino acid residues V274-E398 or T4270-P4487 (Uniprot accession no. F1PHS5), corresponding to actin (∆ABD; orange) and IF (∆IFBD; pink) binding domains. To generate the ∆ABD plectin mutant, we used gRNAs within introns (In) 3–4 and 10–11. To generate the ∆IFBD plectin mutant, we used two gRNAs within the exon (Ex) 32 surrounding the IFBD. For sequences of gRNAs, see the Materials and methods section. **(B and C)** Representative confocal images of cortical F-actin (B) and keratin (C) structures in an apical section of WT, ∆ABD, and ∆IFBD MDCK monolayers immunolabeled for plectin (green) and F-actin (B; magenta) or keratin (C; red). Nuclei, Hoechst (cyan). Images shown in C are also presented in Fig. 5 E. Dashed lines, line scans (right). Boxed areas, ×2 images. Scale bar, 10 and 3 μm (boxed areas). Graphs show line-scan analysis of plectin and F-actin (B) or keratin (C) fluorescence intensities.

To reinforce this conclusion, we generated two MDCK cell lines harboring endogenous plectin with CRISPR/Cas9-engineered deletions between amino acid residues V274-E398 and T4270-P4487, corresponding to the actin (∆ABD) and IF (∆IFBD) binding domains (Fig. 5, C and D and Fig. S4 A). As anticipated, the ΔABD plectin mutant lost its colocalization with cortical F-actin, while it became predominantly distributed along the KFs (Fig. S4, B and C). Conversely, the ΔIFBD plectin mutant colocalized with subplasmalemmal F-actin but failed to form a fine cytoplasmic network superimposed to KFs (Fig. S4, B and C). Strikingly, no circumferential keratin rim was formed in either of the mutant cell lines, indicating the strict requirement of plectin’s interaction with both F-actin and keratin to secure its formation (Fig. 5, E and F). The disappearance of circumferential keratin in ΔABD- and ΔIFBD-expressing lines was accompanied by quantitative keratin bundling and widening of misshaped DSMs (Fig. 5, E–I). The DSM width, a key readout of KF–DSM architecture, was in both mutated cell lines comparable to the enlarged widths measured in KO monolayers (Figs. 1 E and 5 H) and WT monolayers treated either with PST (Fig. 1 E) or LatA (Fig. 5 B).

Finally, we explored how the absence of AB and IFB plectin domains affects epithelial monolayer stability using a dispase dissociation assay. Monolayers grown from mutated cells readily disassembled upon mechanical challenge into a significantly higher number of fragments than WT monolayers (Fig. 5 J). Hence, plectin appears to be instrumental in recruiting keratin to the cortical F-actin to form the circumferential keratin rim, facilitating intercellular adhesion and preserving epithelial integrity.

### Elevated contractility and intercellular tensile stress in plectin KO monolayers

**Figure 6.**
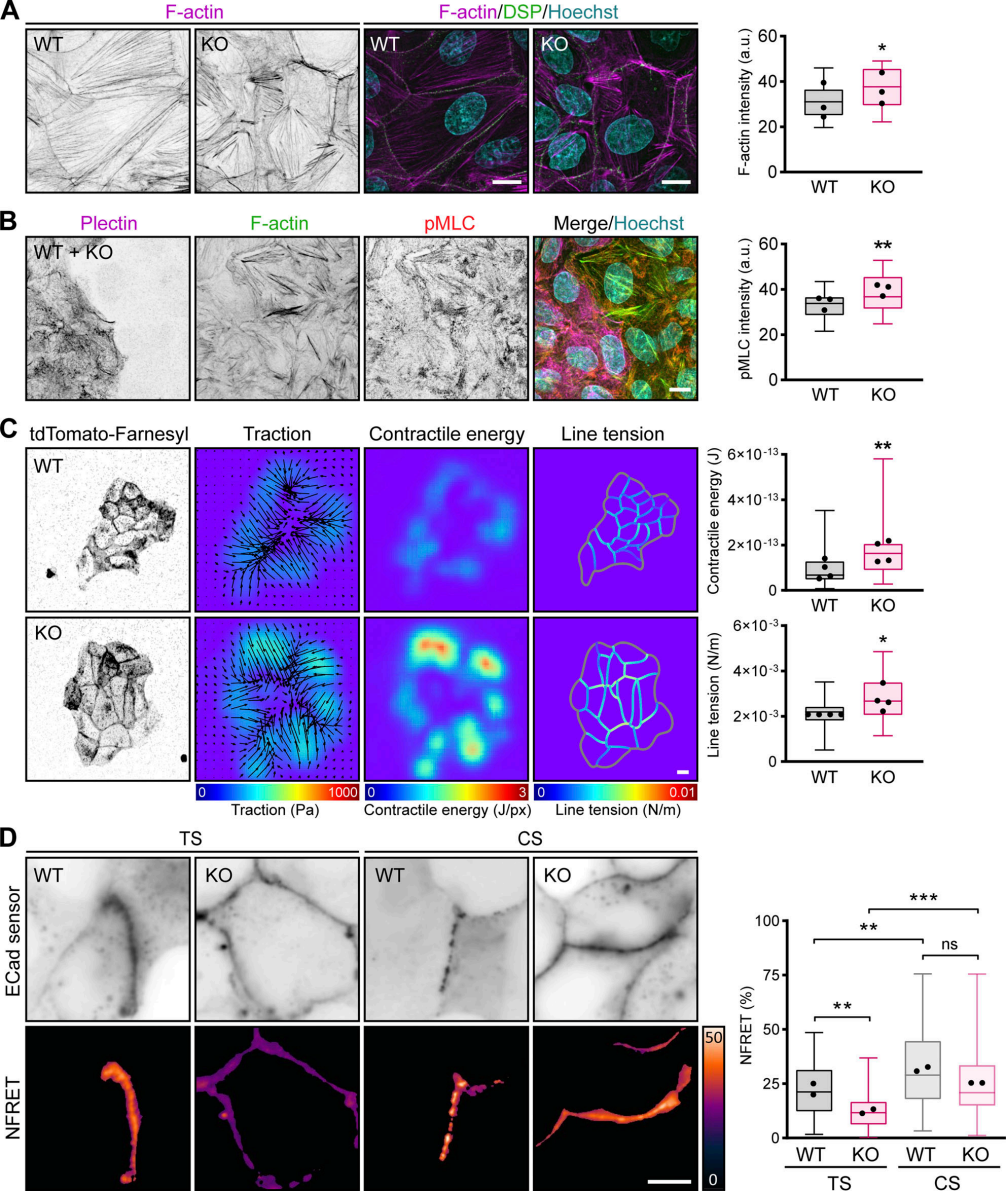
**Plectin ablation accounts for tensile monolayer stress. (A)** Representative confocal images of a 1-μm-thick basal section of WT and KO MDCK monolayers stained for F-actin (magenta) and DSP (green). Nuclei, Hoechst (cyan). Scale bar, 10 μm. The boxplot shows the quantification of F-actin fluorescence intensity. The box represents the 25th and 75th percentile with the median indicated; whiskers reach the last data point; dots, means of independent experiments; *n* = 25 fields of view; *N* = 3. Paired two-tailed *t* test; *, P < 0.05. **(B)** Representative confocal images of a 1-μm-thick basal section of WT + KO monolayer stained for plectin (magenta), F-actin (green), and pMLC (red). Scale bar, 10 μm. The boxplot shows the quantification of pMLC fluorescence intensity. The box represents the 25th and 75th percentile with the median indicated; whiskers reach the last data point; dots, means of independent experiments; *n* = 25 fields of view; *N* = 3. Paired two-tailed *t* test; **, P < 0.01. **(C)** Representative images of WT and KO colonies stably expressing tdTomato-Farnesyl; corresponding pseudocolor spatial maps of traction, contractile energy, and line tension determined by TFM and MSM. Scale bar, 10 μm. The boxplot shows the quantification of contractile energy and line tension. The box represents the 25th and 75th percentile with the median indicated; whiskers reach the last data point; dots, means of independent experiments; *n* = 35 (WT), 40 (KO) cell colonies; *N* = 4. Two-tailed *t* test; *, P < 0.05; **, P < 0.01. **(D)** Representative images of ECad tension (TS) and control (CS) sensors signals (upper panels) and the corresponding FRET index maps (NFRET, lower panels) from WT and KO cells. The boxplot shows the quantification of the NFRET index. The box represents the 25th and 75th percentile with the median indicated; whiskers reach the last data point; dots, means of independent experiments; *n* = 42 (WT TS), 47 (KO TS), 45 (WT CS), and 42 (WT CS) cells; *N* = 2. Significance was calculated from pooled data from two independent experiments. One-way ANOVA Tukey’s multiple comparison test; **, P < 0.01; ***, P < 0.001, ns, P > 0.05.

**Figure S5.**
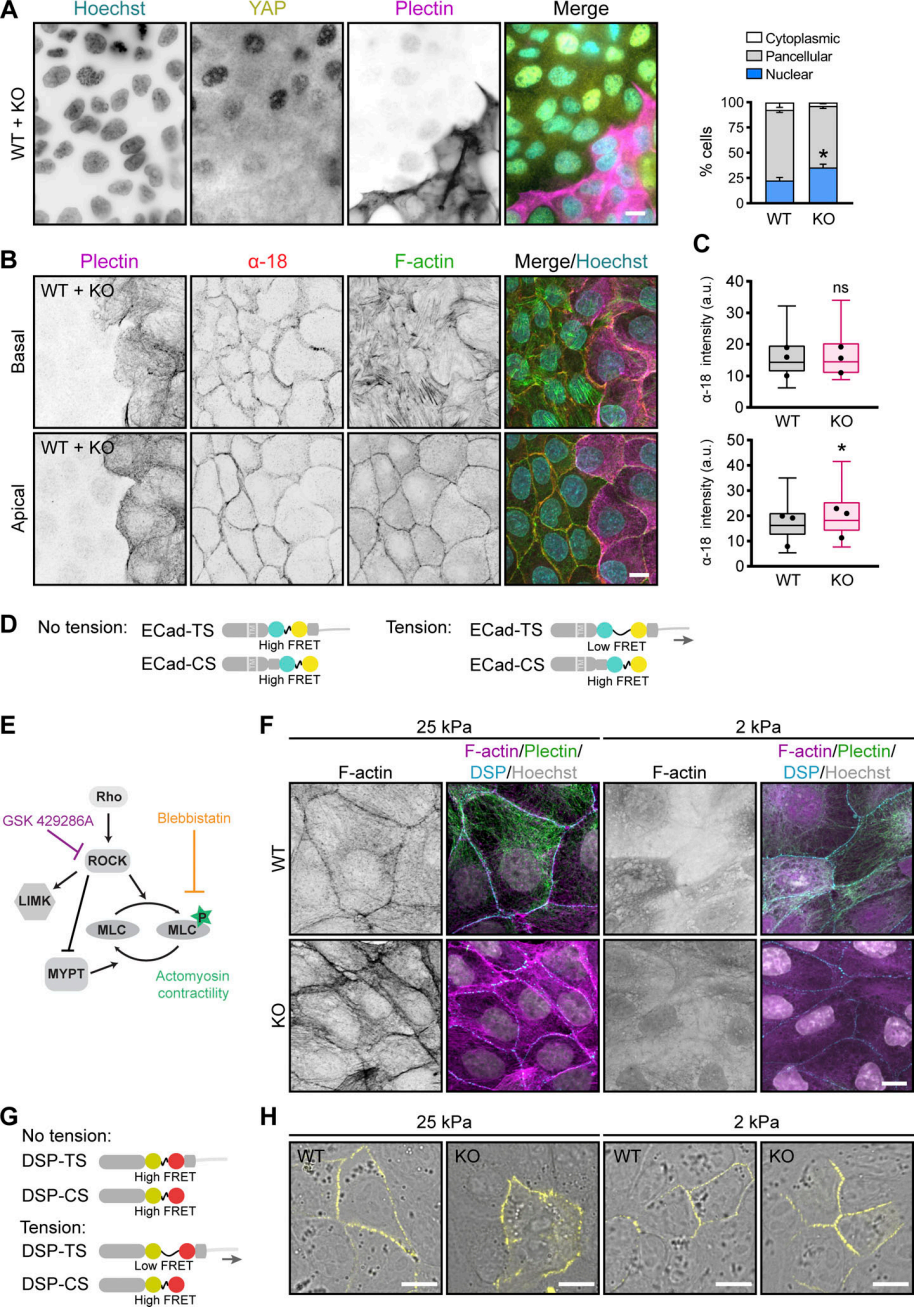
**Increased actomyosin contractility and tensile loading on DSMs in plectin-deficient monolayers. (A)** Representative immunofluorescence images of mixed WT + KO MDCK monolayers immunolabeled for YAP (yellow) and plectin (magenta). Nuclei, Hoechst (cyan). Scale bar, 10 μm. The graph shows the percentage of cells with the YAP signal predominantly nuclear (blue), cytoplasmic (white), or evenly distributed throughout the cell (pancellular; gray). *n* = 652 (WT) and 892 (KO) cells; *N* = 4. Two-tailed *t* test (percentage of nuclear YAP in WT and KO cells was compared); *, P < 0.05. **(B)** Representative confocal images of WT + KO monolayers stained for tension-sensitive α-18 epitope of α-catenin (red), F-actin (green), and plectin (magenta). 1-μm-thick basal (upper panels) and apical (lower panels) planes of the monolayer are shown. Scale bar, 10 μm. **(C)** Quantification of α-18 fluorescence intensities in basal and apical planes of mixed monolayers shown in B. The box represents the 25th and 75th percentile with the median indicated; whiskers reach the last data point; dots, means of independent experiments; *n* = 35 fields of view; *N* = 3. Paired two-tailed *t* test; *, P < 0.05, ns, P > 0.05. **(D)** Schematic representation of ECad tension (TS) and control (CS) sensors. Both sensors comprise the FRET tension module with mTFP (donor) and EYFP (acceptor) fluorophores separated via a flexible nanospring. A tensionless control lacks the β-catenin–binding domain; therefore, cytoskeletal tension reduces FRET in ECad-TS, but not in ECad-CS constructs. **(E)** Schematic representation indicating regulation of actomyosin contractility modulated by contractility inhibitors. GSK inhibits Rho-associated kinase activity, while BLB directly inhibits the myosin-dependent contraction. **(F)** Representative confocal images of WT and KO monolayers on stiff (25 kPa) and soft (2 kPa) collagen-coated hydrogels and stained for plectin (green), DSP (cyan), and F-actin (magenta). Nuclei, Hoechst (gray). Scale bar, 10 μm. **(G)** Schematic representation of DSP I (DSP) tension (TS) and control (CS) sensors. Both sensors comprise the FRET tension module with YPet (donor) and mCherry (acceptor) fluorophores separated via a flexible nanospring. A tensionless control lacks the IF-binding C-terminal region; therefore, tension reduces FRET in DSP-TS, but not in DSP-CS constructs. **(H)** Representative images of DSP-TS signal (yellow) in WT and KO monolayers on hydrogels with high (25 kPa, left) and low (2 kPa, right) stiffness. Scale bar, 10 μm.

Epithelial keratin and actin networks are interrelated and cooperate with intercellular junctions to maintain tensional equilibrium across epithelial sheets (Broussard et al., 2020; Quinlan et al., 2017). To determine if plectin governs epithelial actomyosin networks, we assessed the organization of the actin cytoskeleton. Although cortical F-actin was well established in both types of monolayers (Fig. 3 D), KO cells exhibited more prominent actin stress fibers, evidenced by significantly increased F-actin fluorescence intensity in the basal plane of the cell monolayer (Fig. 6 A). Also, quantitative immunofluorescence microscopy revealed elevated levels of phosphorylated myosin light chain (pMLC), a marker of contractile actomyosin structures, in KO monolayers (Fig. 6 B). Consistent with enhanced actomyosin assembly, higher levels of the transcription factor YAP were found in nuclei of KO cells, indicating activation of tension-dependent signaling pathways in monolayers devoid of plectin (Fig. S5 A). Together, these findings imply that plectin ablation stimulates actomyosin contractility within epithelial sheets.

We then used traction force microscopy (TFM) to measure global force patterns in colonies of MDCK WT and KO cells, and we applied Monolayer Stress Microscopy (MSM [Bauer et al., 2021]) to the TFM data to infer the patterns of mechanical stress within cell colonies. In line with the pronounced actomyosin assembly in KO cells (Fig. 6, A and B), we found higher contractile energy and higher tractions in KO colonies compared with WT colonies (Fig. 6 C). Moreover, MSM analysis revealed increased line tension across cell–cell borders (defined as the force per unit length acting on a segment of a cell–cell border; Bauer et al., 2021) within KO colonies (Fig. 6 C), suggesting that intercellular junctions between KO cells experience higher tensile loading than their WT counterparts.

To test if the increase in actomyosin-generated tension in KO cells led to changes in tension on actin filament–linked AJs, we immunolabeled mixed WT and KO cell monolayers with antibodies against a tension-sensitive α-18 epitope of α-catenin (Yonemura et al., 2010). The intensity of α-18 staining at cell–cell borders within the basal 1-μm-thick section of a cell monolayer did not significantly differ between the areas with WT and KO cells (Fig. S5, B and C). By contrast, we measured a significantly higher α-18 intensity in the apical plane of KO cell areas, which indicates increased tension across AJs and is consistent with the formation of prominent actin stress fibers in KO monolayers (Fig. S5, B and C). We confirmed this finding at the molecular level using a FRET-based tension sensor incorporated into ectopically expressed E-cadherin (ECad-TS; Borghi et al., 2012; Fig. S5 D). The energy transfer was determined exclusively at cell–cell contacts, where ECad-TS and its tensionless control (ECad-CS) were enriched in WT and KO cells to the same levels. Higher FRET in ECad-CS compared with ECad-TS was measured in both WT and KO monolayers, demonstrating that AJs are under tension irrespective of plectin deletion (Fig. 6 D). In agreement with α-18 staining, energy transfer across ECad-TS was significantly lower in KO than in WT monolayers (Fig. 6 D), consistent with increased tension. These results show that plectin-dependent changes in cytoskeletal architecture were associated with increased contractility of KO monolayers and higher tensile loading of AJs.

### Aberrant KF–DSM networks in KO monolayers are under intrinsically generated tensile stress

**Figure 7.**
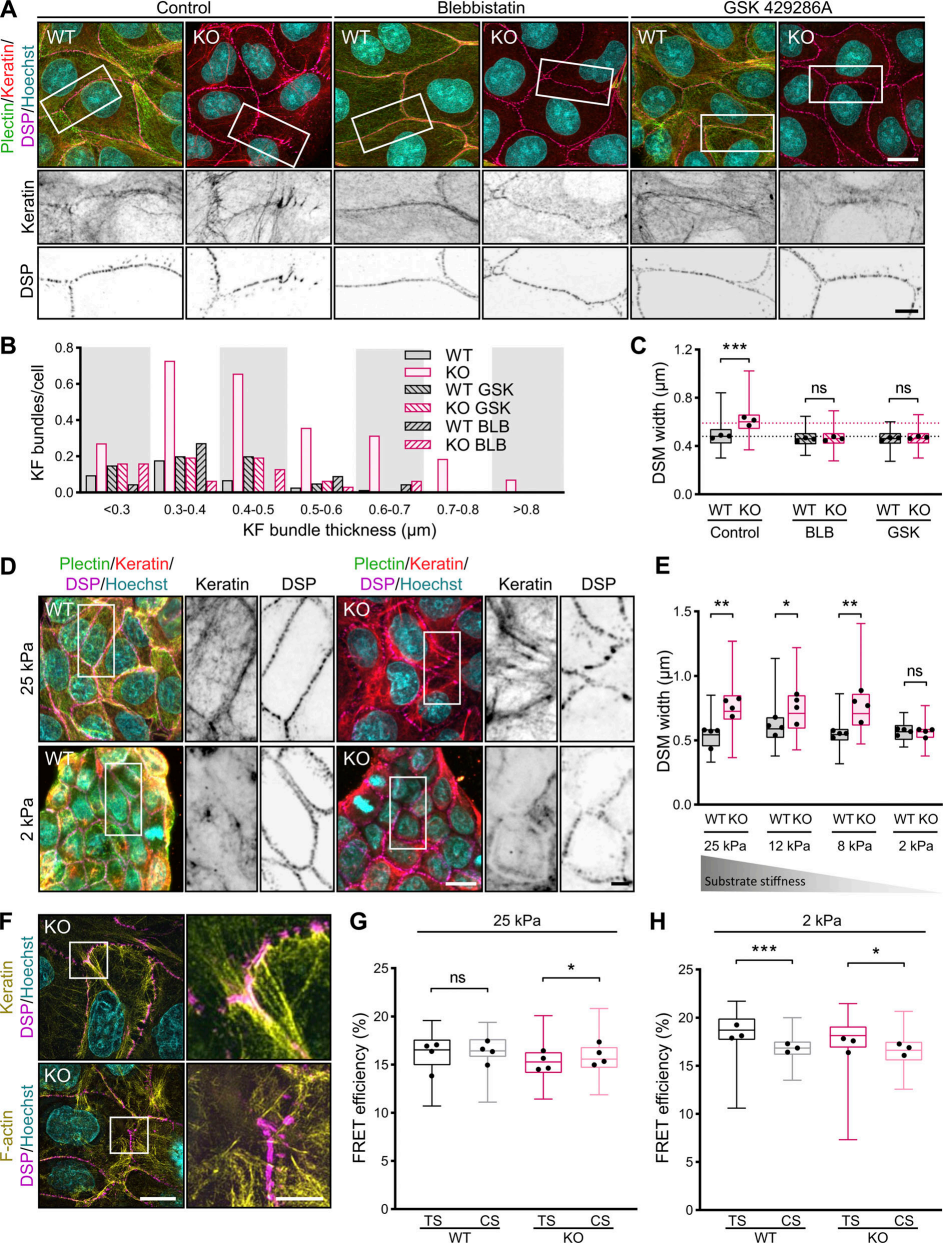
**Collapse of keratin network and tensile loading at DSMs in plectin-deficient monolayers depend on actomyosin contractility. (A)** Representative confocal images of untreated (Control) and BLB (10 μM, 1 h)- or GSK (10 μM, 1 h)-treated WT and KO MDCK monolayers immunolabeled for plectin (green), keratin (red), and DSP (magenta). Nuclei, Hoechst (cyan). Boxed areas, ×2 images. Scale bars, 10 and 5 μm (boxed areas). **(B)** Histogram of KF bundle widths from indicated WT and KO monolayers shown in A. *n* = 73 (WT Control), 70 (KO Control), 22 (WT BLB), 31 (KO BLB), 20 (WT GSK), and 31 (KO GSK) cells; *N* = 3. **(C)** Quantification of DSM widths from indicated WT and KO monolayers shown in A. Dotted lines, median control WT and KO DSM widths. The box represents the 25th and 75th percentile with the median indicated; whiskers reach the last data point; dots, means of independent experiments; *n* = 125 (WT Control), 137 (KO Control), 113 (WT BLB), 118 (KO BLB), 99 (WT GSK), and 109 (KO GSK) DSMs; *N* = 3. One-way ANOVA Tukey’s multiple comparison test; ***, P < 0.001, ns, P > 0.05. **(D)** Representative confocal images of WT and KO monolayers on stiff (25 kPa) or soft (2 kPa) collagen-coated PAA gels and immunolabeled for plectin (green), keratin (red), and DSP (magenta). Boxed areas, ×2 images. Scale bars, 10 and 3 μm (boxed areas). **(E)** Quantification of DSM widths from WT and KO monolayers on PAA gels with indicated Young’s moduli. The box represents the 25th and 75th percentile with the median indicated; whiskers reach the last data point; dots, means of independent experiments; *n* = 134 (WT 25 kPa), 129 (KO 25 kPa), 107 (WT 12 kPa), 130 (KO 12 kPa), 111 (WT 4 kPa), 124 (KO 4 kPa), 105 (WT 2 kPa), and 95 (KO 2 kPa) DSMs; *N* = 4. One-way ANOVA Tukey’s multiple comparison test; *, P < 0.05; **, P < 0.01, ns, P > 0.05. **(F)** Representative SIM images of a KO monolayer stained for keratin (upper panels) or F-actin (lower panels) (both yellow) and DSP (magenta). Note that DSMs bend towards the cell interior in the direction of associated keratin bundles. Boxed areas, ×4 images. Scale bars, 10 and 3 μm (boxed areas). **(G and H)** Quantification of FRET efficiencies for DSP tension (TS) and control (CS) sensors in WT and KO monolayers on stiff (25 kPa; G) or soft (2 kPa; H) collagen-coated PAA gels. Boxplots show the median, 25th, and 75th percentile with whiskers reaching the last data point; dots, means of independent experiments; *n* (G) = 108 (WT TS), 88 (WT CS), 138 (KO TS), 106 (KO CS); *n* (H) = 73 (WT TS), 63 (WT CS), 57 (KO TS), and 60 (KO CS) cell–cell borders; *N* = 4 (G), 3 (H). Significance was calculated from pooled data from four (G) and three (H) independent experiments; Two-tailed *t* test; *, P < 0.05; ***, P < 0.001; ns, P > 0.05.

Given the interdependent nature of actin and keratin cytoskeletons, we hypothesized that increased contractility might contribute to KF–DSM defects in plectin-deficient monolayers. We tested this by experimentally decreasing actomyosin contractility by blocking either myosin II with blebbistatin (BLB) or inhibiting upstream Rho-associated kinase with GSK 429286 (GSK; Fig. S5 E). Both inhibitors abrogated bundling of KFs (Fig. 7, A and B) and dilatation of DSMs (Fig. 7, A and C), which suggested that the KF–DSM phenotypes were myosin sensitive. Restoration of the WT phenotype was particularly conspicuous in DSP-labeled monolayers, as BLB- and GSK-treated KO cells displayed DSMs that were morphologically very similar to those formed by WT cells (Fig. 7 A).

To address the relevance of cell-generated mechanical forces more directly without using small-molecule inhibitors, we compared the morphological response of WT and KO monolayers grown on substrates with Young’s moduli of 25, 12, 8, and 2 kPa using immunofluorescence microscopy (Fig. 7 D). Upon plating on softer matrices (12 and 8 kPa), the DSM width in KO monolayers decreased and reached WT sizes on substrates with low stiffness (2 kPa; Fig. 7, D and E). As expected, this gradual attenuation of the DSM phenotype was accompanied by reduced actomyosin assembly in both WT and KO cells (Fig. S5 F). This suggests that alterations in the KF–DSM networks of KO monolayers are dependent on actomyosin-generated tension.

Externally applied mechanical stimuli, such as monolayer stretching or pulling, have previously been shown to give rise to a tensile loading of DSMs (Price et al., 2018). Our results imply that the contorted, wider DSMs in KO monolayers are exposed to elevated actomyosin-generated tensile stress associated with the collapse of KFs. Moreover, using super-resolution SIM, we found that heavily distorted DSMs bend towards the cell interior in the direction of associated keratin bundles (Fig. 7 F). By contrast, such coalignment was not observed for actin fibers. We then used a FRET-based tension sensor derived from DSP I (DSP-TS; Price et al., 2018; Fig. S5, G and H) to determine whether KF–DSM junctions are under tension (defined as lower FRET efficiency for the TS when compared with CS) in WT and KO monolayers. We quantified DSP-TS and DSP-CS FRET efficiencies using fluorescence lifetime imaging microscopy (FLIM) in cell monolayers grown on hydrogels with high (25 kPa) and low (2 kPa) stiffness. In line with previous observations (Price et al., 2018), in WT monolayers on stiff substrates, FRET efficiencies for DSP-TS were similar to those for DSP-CS, indicating the absence of DSM mechanical loading in WT monolayers. By contrast, DSP-TS FRET efficiency in KO monolayers was significantly lower than that for DSP-CS (Fig. 7 G), indicating tension across DSP I. On soft substrates, where the KO phenotype is comparable to WT cells (Fig. 7, D and E), no tension was detected in WT or KO cells (Fig. 7 H). Instead, the increased FRET efficiencies for DSP-TS might even result from compression of the flexible linker peptide (Paszek et al., 2014; Price et al., 2018), which could potentially indicate a compressive role of the keratin IFs in the force balance (Broussard et al., 2017). These findings underscore the role of actomyosin contractility in tensile loading of DSMs in KO monolayers.

Collectively, these results show that the aberrant keratin cytoarchitecture in KO epithelial sheets is rooted in a disturbed tensional homeostasis. Moreover, they provide evidence that tension-loaded keratin networks exert intrinsically generated tensile stress on DSMs, resulting in their abnormal dilatation and morphology.

## Discussion

**Figure 8.**
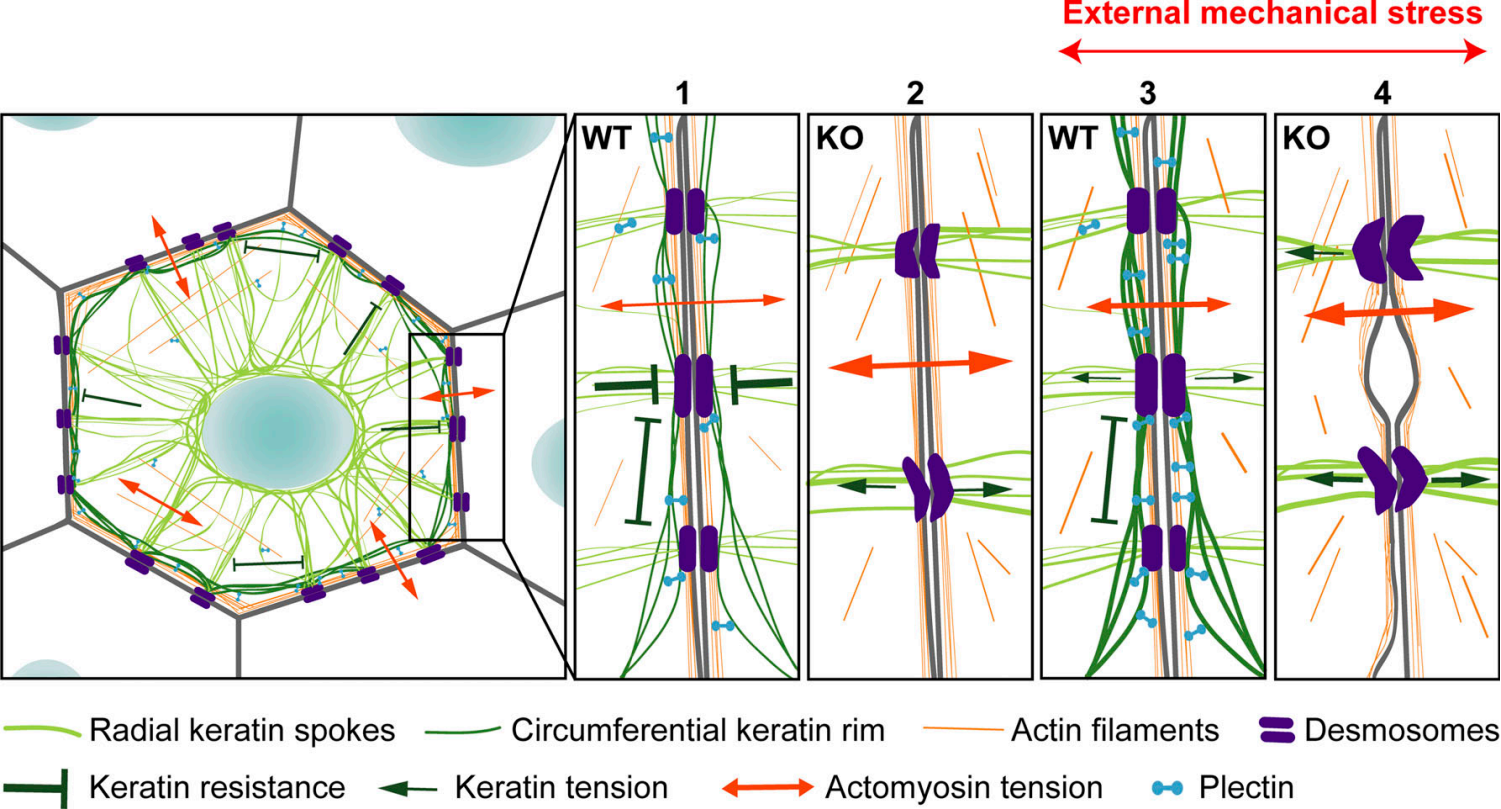
**Model illustrating the role of plectin-mediated cytoskeletal crosstalk for tension distribution and cytoarchitecture in epithelial monolayers.** Cells within epithelia exist in a state of isometric tension, where contractile forces generated by actomyosin (orange) are balanced by compressive elements provided by the keratin network (green). In WT monolayers, the perinuclear KF network extends out into radial spokes (dark green) anchored into DSMs (purple) that are interconnected and evenly distributed by a subplasmalemmal circumferential keratin rim (light green). Radial keratin spokes support the membrane at the sites of DSMs, while the keratin rim provides mechanical resistance along the plasma membrane (1). Plectin deficiency leads to ablation of the circumferential keratin rim. The absence of the keratin rim is associated with the bundling of filaments, leading to a reduction in the number of spokes, which, combined with elevated actomyosin contractility, results in tensile loading of DSMs and internal tensile stress in KO monolayers (2). External mechanical stress in WT monolayers leads to enrichment of subplasmalemmal keratin, which reinforces plasma membrane-bound junctional complexes and provides challenged monolayers with mechanical stability (3). In the absence of plectin-mediated cross-linking of cortical F-actin and keratin, epithelial monolayers fail to adapt efficiently to external mechanical stress, which results in decreased cellular cohesion and epithelial fragility (4).

In this study, we provide evidence that plectin integrates cortical keratin and actin networks to orchestrate the formation of epithelial keratin into a rim-and-spoke configuration (Quinlan et al., 2017). This configuration is reminiscent of a tensegrity structure (Fig. 8), whereby a cross-linking of cytoskeletal components under both compression and tension balances internal tension and stabilizes cellular structures, thus maintaining cell integrity (Ingber, 2003). Taken together, our data suggest that the plectin-controlled circumferential keratin network is required for an even spatial distribution of actomyosin-generated forces between the plasma membrane, DSMs, and cytoplasmic KFs. Plectin depletion mechanically uncouples the individual cytoskeletal network components which leads to the ablation of circumferential keratin, and the redistribution of internal tension drives the bundling of cytoplasmic KFs. These events promote actomyosin assembly and general sheet contractility. Without plectin, the junction-bearing plasma membrane loses mechanical support, and collapsed keratin spokes with actin fibers transfer increased and unevenly distributed cytoskeletal tension to DSMs and AJs. This then leads to a profound destabilization of cell/tissue architecture and the prevention of long-distance force propagation (Na et al., 2009), the latter being required for coordinated cytoskeletal stress adaptation (Ingber, 2003).

We and others have demonstrated that plectin plays a central role in the establishment and maintenance of keratin cytoarchitecture in various isolated cell systems. Plectin depletion was shown to alter the organization of KFs in keratinocytes (Osmanagic-Myers et al., 2006), hepatocytes (Jirouskova et al., 2018), and several epithelial cell lines (Jirouskova et al., 2018; Laly et al., 2021; Moch et al., 2016; Wang et al., 2020). Less dense keratin networks were attributed to the reduction of plectin-mediated orthogonal filament cross-linking (Osmanagic-Myers et al., 2006) and shown to readily disassemble under stress (Jirouskova et al., 2018; Osmanagic-Myers et al., 2006).

Tension-loaded DSP–keratin linkage implies that KFs embedded in aberrant KO networks are under intrinsically generated tension. The organization of keratin in KO monolayers closely resembles the load-bearing *sun-shaped* keratin structures recently found in mechanically stressed subsets of MDCK cell sheets (Latorre et al., 2018). This poses the question of whether loss of orthogonal filament cross-linking alone accounts for the observed keratin disorganization, or whether the collapse of destabilized networks in monolayers occurs only under tensile stress? Intriguingly, morphologically inconspicuous KF–DSM networks (except for the circumferential rim) were reconstituted upon external abrogation of actomyosin contractility with GSK or with BLB. A reduction of cytoskeletal tension upon the cultivation of KO monolayers on soft (2 kPa) hydrogels produced a similar effect. These data also suggest that the loss of cytoskeletal cross-linking disturbs cellular tensional homeostasis and redistributes actomyosin-generated tension. This, in turn, leads to a bundling of KFs, which are devoid of lateral spacing provided by plectin side-arms (Svitkina et al., 1996). As seemingly tensed KFs were also found in β4 integrin-defective EB keratinocytes (Wang et al., 2020), it is conceivable that tensional disequilibrium could also account for the “fragile network” (Russell et al., 2004; Werner et al., 2004) and “sparse network” (Beriault et al., 2012) keratin phenotypes, which were implicated in skin fragility for other EB subtypes. It is noteworthy that aberrant KF–DSM networks are also observed when plectin is disabled either by CRISPR/Cas9 gene ablation or pharmacologically with PST.

To withstand large strains, epithelial sheets undergo dramatic structural alterations that help redistribute mechanical stress and to prevent epithelial damage. Here we show that cyclic stretch of epithelial monolayers initiates a redistribution of the cytoplasmic pool of plectin towards the circumferential cytoskeleton at cell–cell borders. The relocation of plectin was found to be associated with a substantial enrichment and reorientation of KFs at the plasma membrane, where they coaligned with DSMs in a plectin-dependent manner. Although our experimental setup for cell stretching precluded concurrent super-resolution imaging, our data strongly suggest that upon stretch, more abundant plectin integrates newly recruited KFs with the pre-existing circumferential rim and F-actin to reinforce subplasmalemmal networks and mechanically challenged intercellular junctions. Given the extent of plectin-mediated interlinkage of actin and keratin structures, such an adaptive keratin rearrangement likely has effects beyond mere mechanical stabilization and could also contribute to tensile stress relaxation primarily executed by cortical actomyosin (Khalilgharibi et al., 2019).

Plectin-disabled epithelial sheets thus not only harbor aberrant, stress-prone keratin networks, but also fail to reconfigure their cytoskeleton when exposed to mechanical load. Indeed, as shown here, cyclic stretch promoted dysfunctional organization of KFs and resulted in frequent ruptures between adjacent KO cells. Although expanded intercellular spaces occurred only in between individual DSMs, progressive plaque dilatation up to ∼1 μm also implied incidental DSM weakening. This is in line with the prominently enhanced fragmentation of KO monolayers in the dispase-based assay. Further support comes from plectin-deficient in vivo models: upon disruption of keratin networks, DSMs are heavily distorted and unable to prevent epithelial traumas (Jirouskova et al., 2018; Krausova et al., 2021). Hence, we propose that dysfunctional cell cohesion, along with epithelial cell fragility (Jirouskova et al., 2018; Krausova et al., 2021; Osmanagic-Myers et al., 2006) and weakened cell–ECM adhesion (Krausova et al., 2021; Walko et al., 2011), allows strain to act as a mechanotrigger for onset and progression of plectin-associated pathologies.

The recent finding that plectin facilitates recruitment of vimentin IFs to cortical F-actin to control mitotic cortex organization and mechanics (Serres et al., 2020) suggests that the overall concept presented herein is generic (e.g., not restricted to keratin IFs and epithelia). As previous years have provided increasing evidence that dysfunctional biomechanics elicits numerous pathological consequences, we believe that our model provides a coherent framework for a better understanding of various mechanobiological diseases ranging from epithelial fragility to cancer progression and metastasis.

## Materials and methods

### Antibodies

The following primary antibodies were used: rat anti-K8 (DSHB, Krt8; 1:1,000), mouse anti-K18 (Ks 18.04; Progen; 1:1,000), rabbit anti-pan-keratin (z0622; Dako; 1:250), mouse anti-DSG 1+2 (61002; Progen; 1:100), mouse anti-DSP 1+2 (651109; Progen; dilution for immunoblotting [WB] 1:100, for immunofluorescence 1:25), rabbit anti-GAPDH (G9545; Sigma-Aldrich; 1:20,000), mouse anti-PKP2 (MABT394; Merck; 1:100), guinea pig anti-plectin (GP21; Progen, dilution for immunoblotting 1:1,000, for immunofluorescence 1:250), mouse anti-YAP (sc-101199; Santa Cruz Biotechnology; 1:100), and mouse anti-pMLC (3675S; Cell Signaling Technology; 1:200). The mAb (α-18) antibody (1:50) against αE-catenin was a generous gift of A. Nagafuchi (Nara Medical University, Kashihara, Japan). The following secondary antibodies were used: anti-mouse AF-488 (715-545-150; Jackson ImmunoResearch; 1:500), anti-mouse AF-647 (715-605-151; Jackson ImmunoResearch; 1:500), anti-rabbit AF-594 (A11037; Life Technologies; 1:1,000), anti-guinea pig AF-488 (706-545-148; Jackson ImmunoResearch; 1:500), anti-mouse IgG IRDye 680RD (926-68072; LI-COR; 1:20,000), anti-rabbit IRDye 800CW (926-32213; LI-COR; 1:20,000), anti-rat IRDye 800CW (926-32219; LI-COR; 1:20,000), and anti-guinea pig IRDye 680RD (926-68077; LI-COR; 1:20,000). For immunogold labeling, 6 nm colloidal gold donkey anti-guinea pig (706-195-148; Jackson ImmunoResearch; 1:30) was used.

### Cell culture, cDNA constructs, and their expression

MDCK (CCL-34; ATCC) cells (shown in Figs. 1, 2, 3, 4, 5, 6, and 7, Fig. S1, C, D, G, H, and Figs. S2, S3, S4, and S5), immortalized mouse cholangiocytes (Mano et al., 1998; a generous gift of Dr. Y. Ueno, Tohoko University, Sendai, Japan; shown in Fig. S1 B), and human breast epithelial MCF-7 (HTB-22; ATCC) cells (shown in Fig. S1 A; all tested for mycoplasma contamination) were maintained in DMEM (Sigma-Aldrich) supplemented with 10% FBS (Gibco) and 1% penicillin–streptomycin (Sigma-Aldrich) in 5% CO_2_/air humidified atmosphere at 37°C.

Full-length mouse P1 (pGR260), P1a (pGR245), P1b (pGR249), and P1f (pGR258) as well as truncated P1a-8 (pGR161) and P1f-8 (pGR240) cDNA constructs (including 5′ UTRs; Fuchs et al., 1999) encoding fusion proteins with C-terminal GFP were engineered based on published sequences (Fuchs et al., 1999). Transfection was performed using Lipofectamine LTX reagent (Thermo Fisher Scientific). For each transfection reaction (200,000 cells in a six-well plate), 1 μg of plasmid DNA, 1.5 μl of Plus reagent, and 2.6 μl of Lipofectamine LTX reagent were used according to the manufacturer’s instructions. MDCK cells stably expressing tdTomato-farnesyl (Bauer et al., 2021) were prepared using a pLVX-AcGFP-N1 lentiviral vector system. The cDNA of the farnesylated tdTomato fluorescent protein was amplified from tdTomato-farnesyl-5 (58092; Addgene) using a 5′ primer encoding a BamHI site and a 3′ primer encoding a NotI site to replace the AcGFP cDNA in pLVX-AcGFP-N1 (632154; Clontech).

### CRISPR-mediated targeting of *plectin*

Plectin KO cell lines were generated using the CRISPR/Cas9 system, by targeting exon 6 of *plectin* (Plec-205; Ensembl), which encodes the essential ABD and is shared among plectin isoforms. Two distinct gRNA sequences for MDCK cells (5′-AAG​TGA​AGT​TGT​CAC​AGC​GC-3′ and 5′-GAG​GCG​ACC​GTC​ACG​CCA​GC-3′) and cholangiocytes (5′-ACG​GCC​ATC​GCG​CCA​ACT​GG-3′ and 5-TTC​ACC​ACC​AGT​TGG​CGC​GA-3′) were designed. To generate plectin mutant MDCK lines, a pair of gRNA sequences surrounding the targeted regions was designed for each mutation. To generate ΔABD mutant cell lines, gRNA sequences located in the intron 3–4 (5′-CAA​GAG​AGC​GGG​ACG​TAA​TC-3′) and intron 10–11 (5′-GGC​TGA​GTG​AGG​CCG​TCC​AA-3′) were used. To generate ΔIFBD mutant cell lines, gRNA sequences located within exon 32 surrounding the IFBD (5′-CAG​CAG​GCA​CAA​GCC​CGT​CT-3′ and 5′-CGG​TGA​ACG​CTT​CCC​CGT​CA-3′) were designed. gRNA sequences were synthesized and subcloned into a modified version of a px330-Cas9-Venus vector using the BbsI restriction site (Jirouskova et al., 2018), where Cas9 was fused to monomeric Venus as a selection marker. Cells were transiently transfected using Lipofectamine LTX with Plus Reagent (Life Technologies) according to the manufacturer’s instructions. Cells were kept with transfection complexes for 48 h, followed by FACS. Single-cell clones were derived by dilution cloning in 96-well plates, and plectin knockout or mutation was confirmed by DNA sequencing and immunoblot analysis.

### Drug treatments

PST (Meier et al., 2017) was used at concentrations of 4, 8, and 16 μM for 4 h. Stocks were dissolved in dimethylsulfoxide and diluted in complete medium. For depolymerization of cytoskeletal components, LatA (actin cytoskeleton) was used at 1 μM concentration for 30 min or NCD (microtubules) at a concentration of 10 μM for 1 h. For actomyosin inhibitor treatments, BLB was used at a concentration of 10 μM for 1 h, and GSK 269962A was used at a concentration of 10 μM for 1 h.

### Protein extraction and immunoblot analysis

Cells were rinsed with PBS and lysed in RIPA buffer (20 mM Tris pH 7.4, 150 mM NaCl, 0.1% SDS, 0.5% Na-Deoxycholate, and 1% Triton X-100) supplemented with protease and phosphatase inhibitors (Roche). Protein concentrations were determined using a BCA Protein Assay Kit (Thermo Fisher Scientific). Proteins were separated by SDS-PAGE and transferred to nitrocellulose membranes. Membranes were incubated with 5% BSA in PBST to block nonspecific antigen interactions and subsequently incubated with primary and secondary antibodies. Signals were detected and quantified using an Odyssey imaging system (LI-COR).

### Immunofluorescence microscopy

Unless stated otherwise, 200,000 cells were plated on collagen-coated coverslips (18 mm, No. 1.5) in a six-well plate well and kept in a CO_2_ incubator for 48 h to form a freshly confluent monolayer. Cells were fixed either with ice-cold methanol for 1 min (for keratin staining) or with 4% PFA for 10 min (for F-actin staining). When PFA was used for fixation, cells were subsequently permeabilized with 0.2% Triton X-100 for 10 min. Nonspecific antigen interactions were blocked with 5% BSA in PBST for 1 h. Incubation with primary antibodies was performed either for 3 h at RT or overnight at 4°C in a humidified chamber. Coverslips were incubated with secondary antibodies, together with Hoechst 33258 (Sigma-Aldrich) and, when indicated, also with fluorescently labeled phalloidin (Invitrogen) for 1 h at RT in the dark.

### Image acquisition and processing

Immunofluorescence images were acquired at RT using a Leica DM6000 wide-field microscope (Leica Microsystems) with a Leica DFC 9000 sCMOS camera and an APO 100×/1.4 NA immersion oil objective with Type F Immersion liquid (Leica Microsystems). The acquisition was performed using LasX software. Immunofluorescence confocal images were acquired at RT using a Leica TCS SP8 confocal fluorescence microscope (Leica Microsystems) with photomultiplier tubes, supersensitive hybrid detectors, and an HC PL APO 63×/1.4 NA immersion oil objective, using Type F Immersion liquid (Leica Microsystems). The acquisition was performed using LasX software. Super-resolution fluorescence SIM images were acquired at RT using a DeltaVision OMX V4 microscope with a Blaze SIM module (GE Healthcare Life Sciences) and a Photometrics CoolSANP HQ camera and PLAN APO N 60×/1.42 oil immersion objective with immersion oil (refractive index 1.516 or 1.518; GE Healthcare). The acquisition was performed using OMX Acquisition control software. The raw images taken were reconstructed and registered with proprietary SoftWoRx software equipped with a structured illumination image reconstruction module using optical transfer function optimized for green/red emission and an individual channel image registration module. Super-resolution fluorescence STED images were acquired at RT using a Leica TCS SP8 STED3x microscope (Leica Microsystems) with photomultiplier tubes, hybrid detectors, and an HC PL APO 100×/1.4 NA oil STED objective with type F immersion liquid (Leica Microsystems). The acquisition was performed using LasX software. STED imaging was followed by deconvolution in Huygens Pro software (Scientific Volume Imaging). Image sampling was set to be close to the Niquist rate (typically, for a standard confocal imaging, voxel size was ∼60 × 60 × 200 nm). Unless stated otherwise, representative maximal projections of z-stacks, acquired from the whole cell volume (usually imaged from the first to the last Z-plane where DSP signal was well visible, typically 7–10 slices) are shown. Postacquisition processing (such as formation of maximal projections, linear adjustments of brightness and contrast, inversion of grayscale images, changes in lookup tables, and cropping or resizing) was performed using an open-source ImageJ image processing package (Schindelin et al., 2012).

### Transmission EM

Cells were grown on collagen-coated 10 mm coverslips (or elastic silicone chambers for stretching) until reaching confluency (typically 48 h after plating 200,000 cells per well of a six-well plate). Monolayers were fixed in 2.5% glutaraldehyde and 2% PFA in Sorensen’s buffer. Samples were postfixed with 1.5% OsO_4_ in Sorensen’s buffer, contrasted with 1% uranyl acetate in 50% ethanol, and dehydrated through a graded ethanol series followed by propylene oxide. Dehydrated samples were embedded into a Quetol 651 resin (Electron Microscopy Sciences). Ultrathin sections were placed on a Formvar film on 3-mm TEM slots, contrasted by uranyl acetate and Reynolds’ lead citrate, and visualized with a JEM-1400 FLASH microscope (Jeol) equipped with a FLASH 2k × 2k CMOS camera at 80 kV. Immunogold labeling with plectin antibody was performed as previously described (Eger et al., 1997). Briefly, cells cultivated on coverslips were rinsed with 200 mM Hepes (pH 7.4, 1.8 mM CaCl_2_, 1 mM MgCl_2_) supplemented with a protease inhibitor cocktail (Sigma-Aldrich). After prefixation in Hepes buffer (0.1% glutaraldehyde, 0.1% Triton X-100) and reduction of free aldehyde groups (0.5 mg/ml NaBH_4_), cells were blocked with 0.2% gelatin. For immunolabeling, cells were incubated with anti-plectin antibodies (6 h at 4°C) and 6 nm gold particle–conjugated secondary antibodies (14 h at 4°C). Samples were then subsequently postfixed in 1% glutaraldehyde and 1% OsO_4_. Postfixed specimens were further dehydrated in an ethanol series and embedded in Quetol (72 h at 60°C). Ultrathin sections were contrasted with uranyl acetate/lead citrate and viewed under a Jeol JEM-F200 transmission electron microscope at 200 kV. Images were acquired with a 4k × 4k TVIPS TemCam – XF416 cooled CMOS camera.

### Cell stretching

Stretching experiments were carried out using a custom-made stretcher device (Kah et al., 2021). Cells were plated on flexible polydimethylsiloxane (Sylgard) membranes with 4 cm^2^ internal surface, activated with 0.5 mg/ml Sulfo-SANPAH (Thermo Fisher Scientific) treatment under UV light for 5 min, and coated with 50 μg/ml collagen for 1 h at 37°C. 250,000 cells/cm^2^ were seeded on the polydimethylsiloxane membrane in 1.5 ml of DMEM and incubated overnight. Uniaxial cyclic stretch was applied using a custom-made stretcher device (Kah et al., 2021) for 6 h at 20% stretch amplitude in an incubator under standard cell culture conditions (37°C, 5% CO_2_, 95% humidity).

### Dispase-based dissociation assay

Cells were seeded on 10 mm coverslips in a six-well plate (500,000/well) and grown to confluency. 24 h after confluency, cell monolayers were detached by incubating with 2.4 U/ml dispase (D4693; Sigma-Aldrich) solution for 40 min at 37°C. Detached monolayers were carefully transferred to 15-ml conical tubes and subjected to mechanical stress by inversion (10×). Monolayer fragments were transferred into clean six-well plates and counted.

### Traction force microscopy and monolayer stress microscopy

50,000 tdTomato-farnesyl–expressing MDCK cells were seeded on collagen-coated 7.85% polyacrylamide (PAA) gels (Young’s modulus 49.8 kPa, thickness 300 µm), with embedded 1-μm green fluorescent beads (Invitrogen; Pelham and Wang, 1998; Raupach et al., 2007). The cells were incubated under standard growth conditions for 48 h to form small epithelial colonies. TFM imaging was performed using a Dragonfly (Andor Technologies) spinning disc confocal microscope with a Zyla 4.2 PLUS sCMOS camera and an APO 20×/0.75 NA multi-immersion objective with water used as the immersion medium. During measurements, cells were kept in a live-cell imaging chamber (37°C and 5% CO_2_ in a humidified atmosphere; OKOlab). Image acquisition was performed using Andor software. Cell tractions and line tensions were computed using pyTFM software (Bauer et al., 2021) from images taken before and after trypsin-based cell removal.

### ECad tension sensor measurements

MDCK cells were transiently transfected with tension (TS) and control (CS) E-Cad sensors (Borghi et al., 2012), containing an mEYFP-mTFP FRET module (Epoch Life Science) inserted into canine E-cadherin cDNA (between V742 and K743 [TS] or following residues 1–768 [CS]). Transfected cells were imaged after 48–72 h in phenol-free DMEM in a live-cell imaging chamber (37°C and 5% CO_2_ in a humidified atmosphere; OKOlab), using a Nikon H-TIRF microscope, equipped with a Nikon CFI Plan APO 60× NA 1.49 oil objective with type F immersion liquid (Leica Microsystems), an EM CCD Andor iXon Ultra DU888 camera (Andor Technologies), and a W-View Gemini image splitter (Hamamatsu Photonics).

Fluorescence images were analyzed using the PixFRET plug-in (Feige et al., 2005) from ImageJ software (Schindelin et al., 2012). The raw fluorescence intensity from the FRET channel (IFRET) was corrected for spectral bleed-through of the acceptor (*BTacceptor*) and donor (*BTdonor*) pixel by pixel to yield the corrected FRET intensity $NFRET=\frac{IFRET-BTdonor\times Idonor-BTacceptor\times Iacceptor}{\sqrt{Idonor\times Iacceptor}}\times 100.$ Manual intensity thresholding of the acceptor channel was used to segment the cell–cell contact regions. NFRET was then averaged over the segmented cell–cell contacts, and data from two independent experiments were plotted.

### DSP tension sensor measurements

MDCK cells were plated on imaging dishes with hydrogels of defined stiffness (stiff, 25 kPa; or soft, 2 kPa; Matrigen, SV3520-EC-25/2 PK) and transfected with tension (TS) and control (CS) DSP sensors (Price et al., 2018), containing the YPet-F40-mCherry module (DPI-TS, 118725; Addgene; DPI-CS, 118726; Addgene) or the corresponding donor-only controls containing fluorescently dead mCherry (Y72L). 48–72 h after transfection; cells were imaged in DMEM without phenol red in a live-cell imaging chamber (37°C and 5% CO_2_ in a humidified atmosphere; OKOlab). FLIM measurements were acquired using a TCS SP8 (Leica Microsystems) inverted confocal microscope, equipped with a white-light laser, HydraHarp400 (Picoquant), and an HC PL APO CS2 63×/1.2 NA water immersion objective with water used as an immersion liquid. Image acquisition was performed using LasX software. To analyze time-correlated single-photon counting (TCSPC)-FLIM data, custom-written MATLAB programs and software were used (Price et al., 2018). The DSM signal was extracted from the intensity image by manually drawing masks and subsequently blurring the image and isolating connected bright regions. To determine the fluorescence lifetime, a monoexponential decay was fitted to the summed photon count time trace, using custom-written MATLAB software (Price et al., 2018). The FRET efficiency (E) was calculated using the following formula: $E=1-\frac{\tau DA}{\tau D},$ with τDA representing the lifetime of the donor in the presence of an acceptor and τD representing the mean of a donor-only lifetime. The mean donor-only lifetime was calculated from matched constructs with a mutated acceptor.

### Statistical analyses

All graphs and statistical tests were performed using GraphPad Prism (GraphPad Software). In the boxplots, the box represents the 25th and 75th percentile with the median indicated; whiskers reach the last data point; dots indicate means of independent experiments. The number of independent experiments (*N*) and data points (*n*) is indicated in each of the figure legends. Normally distributed parametrical data were analyzed by two-tailed unpaired student’s *t* test. A comparison between multiple groups was performed using one-way ANOVA with Tukey’s multiple comparison test. Data distribution was assumed to be normal, but this was not formally tested. Statistical tests used are specified in the figure legends. Statistical significance was determined at the level of *, P < 0.05; **, P < 0.01; ***, P < 0.001; ns, P > 0.05.

### Online supplemental material

Fig. S1 shows the disrupted cytoarchitecture of KF–DSM networks in MDCK cells, cholangiocytes, and MCF-7 cells upon plectin inactivation. Fig. S2 shows stretch-induced changes, expression of P1f-8-EGFP and NCD treatment in MDCK cell monolayers. Fig. S3 shows re-expression of EGFP-tagged plectin isoforms and LatA treatment in MDCK cell monolayers. Fig. S4 shows CRISPR/Cas9-mediated ablation of actin and IF binding domains. Fig. S5 shows increased actomyosin contractility and tensile loading on DSMs in plectin-deficient MDCK cell monolayers.

## Supplementary Material

SourceData F1 contains original blots for Fig. 1.

SourceData F5 contains original blots for Fig. 5.

SourceData FS1 contains original blots for Fig. S1.

SourceData FS2 contains original blots for Fig. S2.
