# Supplementary figures and images for: Plectin-mediated cytoskeletal crosstalk controls cell tension and cohesion in epithelial sheets
Source: J Cell Biol. 2022 Feb 9;221(3):e202105146. doi: 10.1083/jcb.202105146 (PMC8932528; doi:10.1083/jcb.202105146)

Supplementary Figure 1C

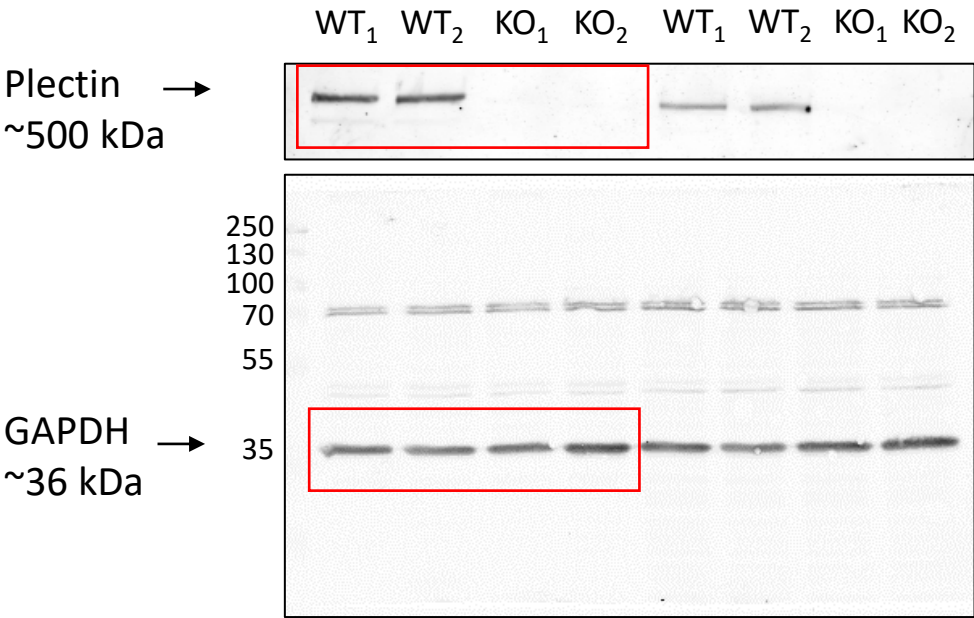

Supplementary Figure 1E

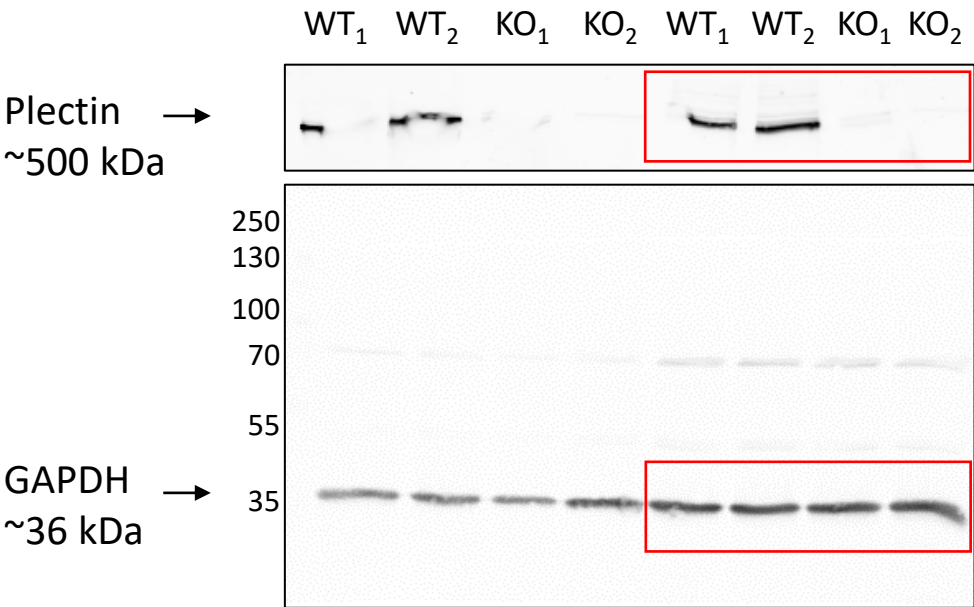

Supplementary Figure 1H

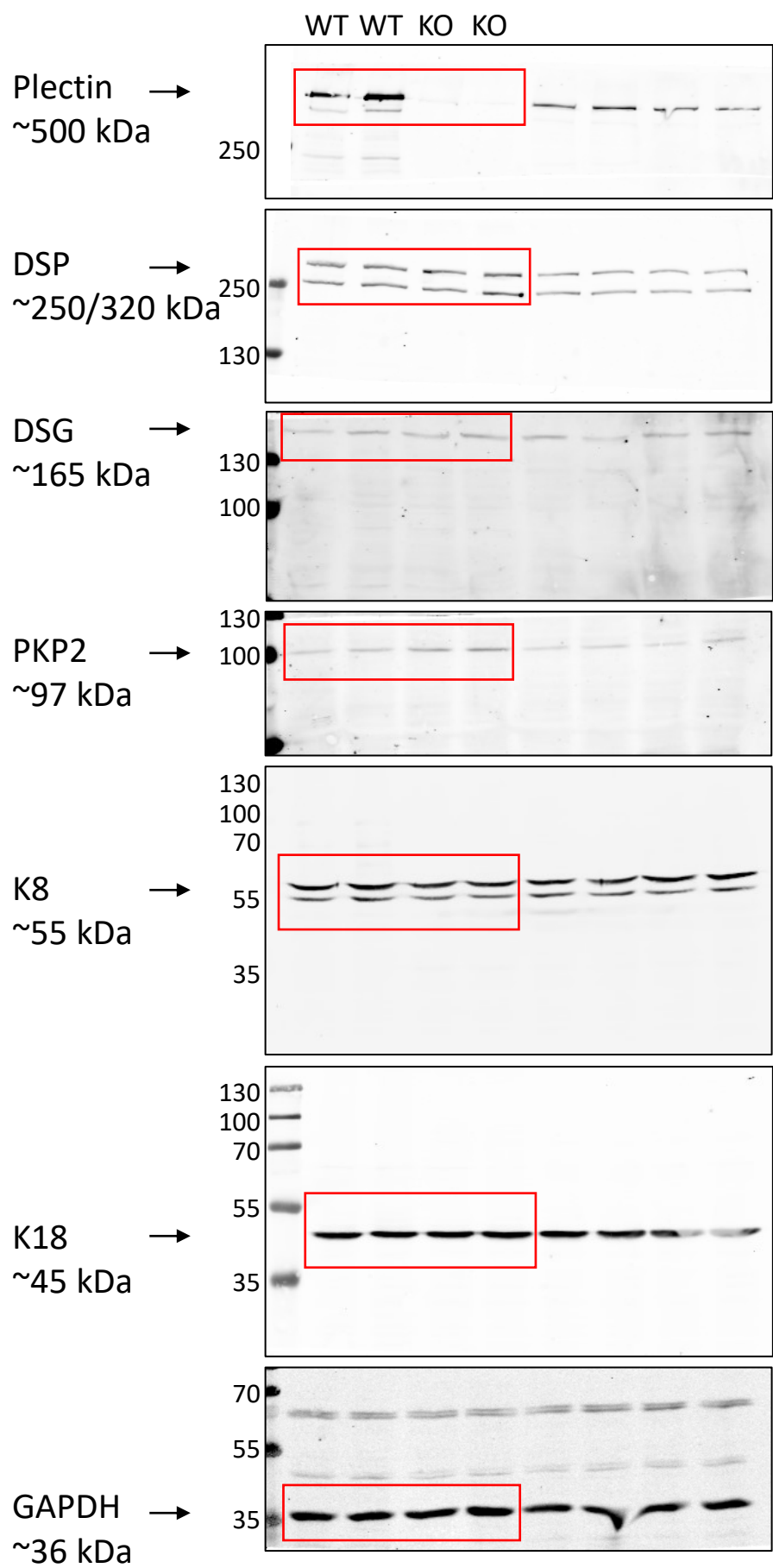

Supplement: SourceData F1 — contains original blots for Fig. 1. [file JCB_202105146_SourceDataF1.pdf]

Figure 5

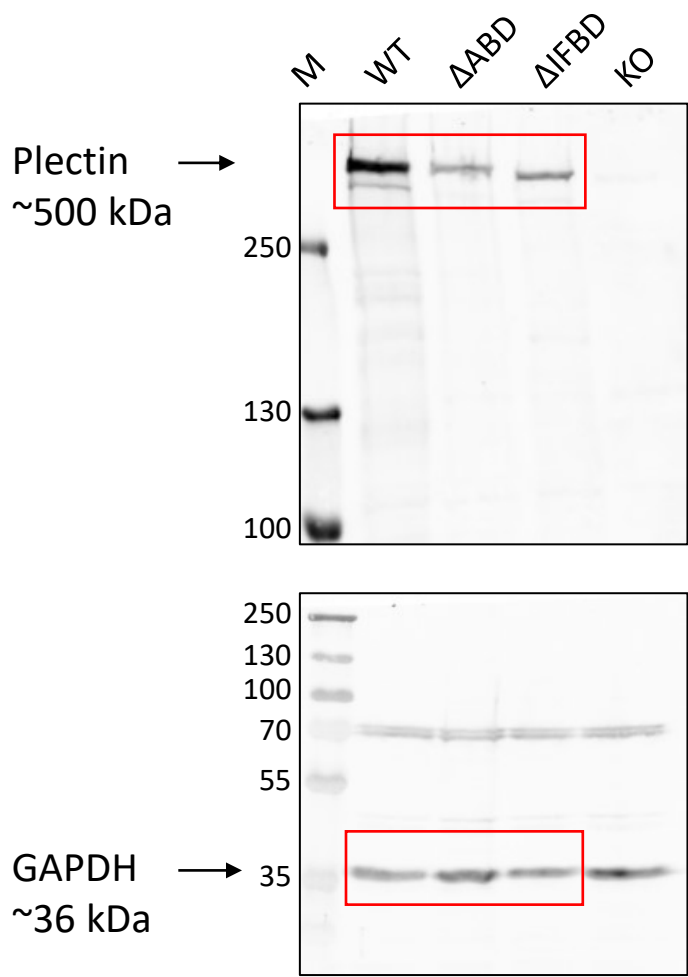

Supplement: SourceData F5 — contains original blots for Fig. 5. [file JCB_202105146_SourceDataF5.pdf]

# Supplementary Figure 2A

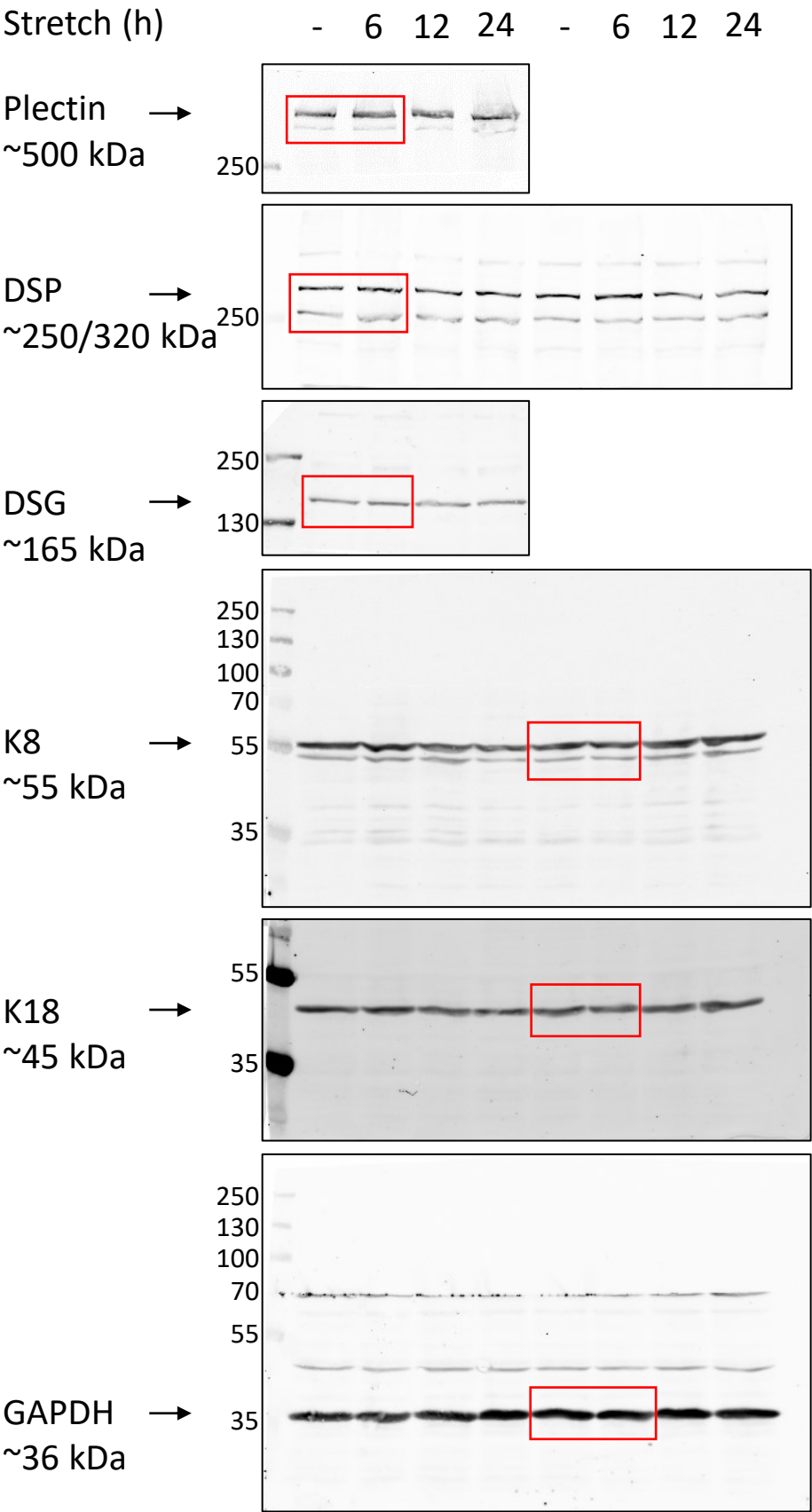

Supplement: SourceData FS2 — contains original blots for Fig. S2. [file JCB_202105146_SourceDataFS2.pdf]
